# Recent Advances in Catalytic and Technology-Driven Radical Addition to *N*,*N*-Disubstituted Iminium Species

**DOI:** 10.3390/molecules28031071

**Published:** 2023-01-20

**Authors:** Sylvain Oudeyer, Vincent Levacher, Hélène Beucher, Jean-François Brière

**Affiliations:** Normandie Univ, UNIROUEN, INSA Rouen, CNRS, COBRA, 76000 Rouen, France

**Keywords:** radical, dipole, catalysis, photoredox, electrosynthesis

## Abstract

Recently, radical chemistry has grown exponentially in the toolbox of organic synthetic chemists. Upon the (re)introduction of modern catalytic and technology-driven strategies, the implementation of highly reactive radical species is currently facilitated while expanding the scope of numerous synthetic methodologies. In this context, this review intends to cover the recent advances in radical-based transformations of *N*,*N*-disubstituted iminium substrates that encompass unique reactivities with respect to imines or protonated iminium salts. In particular, we have focused on the literature concerning the dipole type substrates, such as nitrones or azomethine imines, together with the chemistry of N^+^-X^−^ (X = O, NR) azaarenium dipoles, which proved to be very versatile platforms in that field of research. The *N*-alkylazaarenium salts were been considered, which demonstrated specific reactivity profiles in radical chemistry.

## 1. Introduction

While radical chemistry has been an active and highly creative field of research for a long time, the last decade has testified an increased amount of published work highlighting a renewed attention of the synthetic chemist community and even some, a priori, non-specialists to this field [1]. Needless to say, the unique and complementary selectivity of radical processes versus ionic-based transformations, together with the opportunity to perform functional group-tolerant—or even protective group-free—transformations, has elicited curiosity of organic chemists in quest of more expeditious syntheses. However, the evolution of the initial use of stochiometric, and sometimes toxic radical reaction promoters to catalytic radical sequences has established the foundations to allow a larger community to embrace radical chemistry as a potentially more sustainable strategy for the construction and functionalization of complex molecules, even in late stages of a synthetic plan. The progresses in catalytic metal-based redox approaches first contributed to this evolution [2], but the more recent (re)emergence of technology-driven single-electron transfer (SET) processes has accelerated new discoveries in that field with, at the forefront, visible light photoredox catalysis [3]. Even though the real classification of electrosynthesis as a catalytic methodology deserves discussions with regard to the tactic used [4,5,6], the modern evolution of organic electrochemistry and electrocatalysis, in connection with the availability of user-friendly apparatus [7], has boosted the technique as a tool of choice for radically initiated processes as well [8,9,10,11].

**Figure 1:**
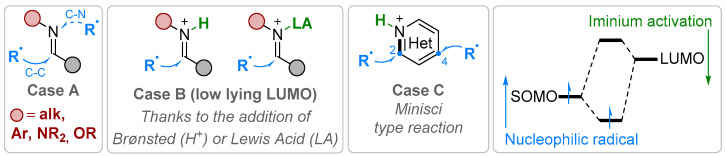
Radical addition to imine motifs and derivatives: general aspects.

**Figure 2:**
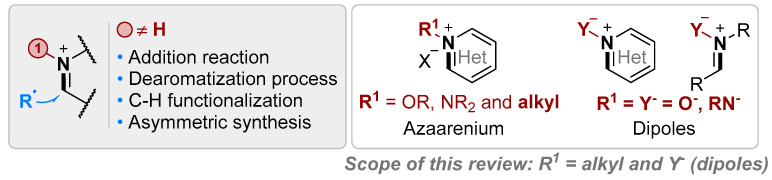
Scope of this review.

This wave of radical chemistry has also led to a large array of investigations aiming at getting insight into reactivity issues while improving the scope and selectivity of functional group transformations. In that domain, the formal addition reaction to imines through radical processes has met an enormous amount of studies due to the ubiquitous presence of amine motifs within naturally occurring and bioactive compounds [12,13,14]. Generally speaking, the imine functional group undergoes a C–C bond formation (Figure 1, case A) albeit an addition to the nitrogen atom for C-N bond formation is also encountered in few cases, depending on stereoelectronic effect and whether the reaction is performed under an inter- or intramolecular fashion [13]. The imine derivatives such as hydrazines, alkoxyamines, precursors with an electro-withdrawing group on the nitrogen atom, etc., were preferentially used due to favorable stabilization of aminyl radical intermediates obtained after the first addition step. Furthermore, it has been shown that these imine compounds could be activated by means of, at least, a stochiometric amount of a Brønsted or Lewis acid to accelerate the regioselective C–C bond construction through iminium intermediates, and asymmetric sequences were proven when these acids were chiral (Figure 1, case B, LA* or H^+^*). If one recognized an iminium motif embedded within a heterocyclic architecture, then the so-called Minisci reaction occurs on protonated azaarenes [15,16]. This strategy has met important successes for the formal C–H functionalization event, albeit facing the C2 versus C4 regioselectivity issue (Figure 1, Case C). The addition of carbon-centered radicals to iminium or cationic azaarene has been discussed by Opatz and colleagues in an insightful review showing how orbital interactions play a key role in the efficiency of radical addition processes both in terms of rate and regioselectivity [14]. In other words, the interaction between the SOMO (singly occupied molecular orbital) species, considered as nucleophiles, and the low lying LUMO (lowest unoccupied molecular orbital) of iminium species is energetically favorable under the activation with either Brønsted or Lewis acids (Figure 1). Following a similar activation model (Figure 2), several addition sequences of radical species to *N-*alkoxy and *N*-amine azaarenium platform molecules (R^1^ = OR, NR_2_) emerged in the literature as powerful functionalization strategies showing that the regioselectivity issue highly depends on the architecture and substitution pattern of the heterocycles. This field of research was covered by very instructive recent reviews [17,18,19,20,21].

In this review, we intend to cover the recent evolution of the catalytic and technology-driven addition of radical species to iminium and azaarenium compounds having a trisubstituted nitrogen atom (Figure 2, R^1^ ≠ H). In a complementary point of view of what has been covered in the literature, we will hereby focus on both dipole derivatives (N-R^1^ with R^1^ = Y^−^) and *N*-alkyl azaarenium salt, which has never been covered in a unified fashion despite some sporadic examples being found in previous reviews on pyridine derivatives [17,18,19,20,21,22,23]. We essentially cover the literature from 2015, since the Opatz’s review has already discussed this field of research before [14]. Worthy of note, the metal-catalyzed C–H bond functionalization involving a radical process, which is triggered by a metal insertion into the C–H bond, is outside the scope of this review. Likewise, the in situ formation and use of iminium through the oxidation of the corresponding amine as starting material, for instance, will not be considered herein.

For the sake of comparison, this review is organized by type of iminium structure to highlight the differences and complementarities of both the synthetic strategy and the technology employed for a given architecture with a focus on catalytic cycle mechanisms whenever possible.

## 2. Dipole Type Iminium Species

### 2.1. Nitrones

**Figure 3:**
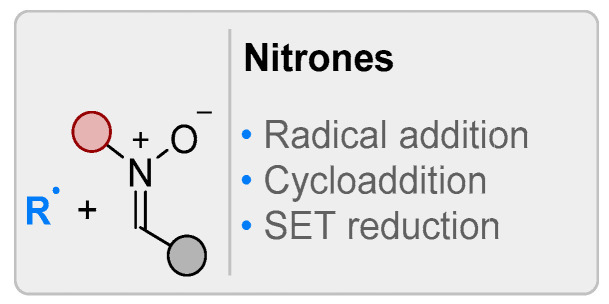
Radical-based transformations of azomethine imines.

Nitrones are versatile platform molecules involved in cycloaddition or nucleophilic addition reactions of polar reagents [24]. Nitrones are known to be potent radical trap leading to rather stable aminoxyl radical intermediates sometimes used for mechanistic investigations. Early contributions showed that stochiometric metal-based radical transformations are also allowed, particularly making use of samarium as a SET reagent [24]. In the mid-2010s, photochemical or photoredox catalytic strategies appeared and opened new ways to functionalize these types of dipoles (Figure 3).

**Scheme 1:**
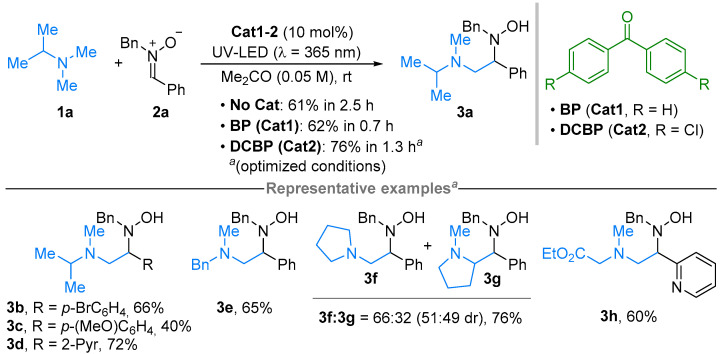
Photoredox-catalyzed reaction of aliphatic α-aminoalkyl derivatives with nitrones.

The photoirradiation of nitrones usually leads to photoexcited counterparts that cyclize into oxaziridine. In order to overcome this pathway, Itoh, Suga and colleagues proposed in 2015 a photoinduced electron transfer (PET) process of a photoexcited nitrone **2** and a tertiary amine **1** (Scheme 1). Upon irradiation with a UV-LED, in acetone as solvent, the authors demonstrated a smooth addition of the amine **1a** to **2a**, leading to the formation of adduct **3a** in 61% yield after 2.5 h. Interestingly, in the presence of a catalytic amount of benzophenone (BP, 10 mol%) as a photosensitizer, the addition reaction was accelerated and took place with **2a** in only 0.7 h to deliver 3a in 62% yield. A screening of conditions revealed the 4,4′-dichlorobenzophenone (DCBP) as a more efficient photosensitizer allowing an improved yield of 76% (**3a**) after 1.3 h.

Under these optimized conditions, the amino alkylation reaction was successfully applied to various nitrones with aryl (**3b**–**c**) or heteroaryl (**3d**) moieties (Scheme 1). Importantly, as testified with *N*-benzyl products **3e**, the selective generation and reaction of the primary α-aminoalkyl radical was observed according to Lewis’s stereoelectronic rules for stilbene–amine mixture [25], although this selectivity issue was more balanced with cyclic substrates in light of the obtained mixture of products **3f** and **3g** with a pyrrolidine structure. The use of amine bearing an ester group was also tolerated for the C–C bond formation towards the construction of **3h**.

**Scheme 2:**
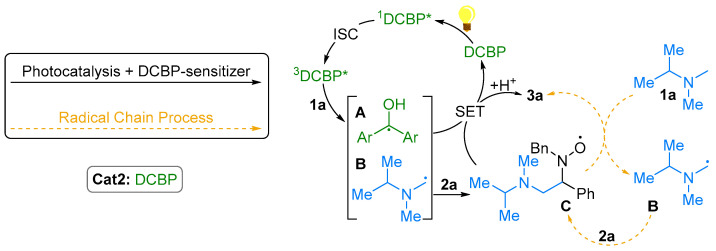
Proposed mechanism for the photoredox-catalyzed reaction of α-aminoalkyl derivatives with nitrones.

At first glance, considering that the direct irradiation of the nitrone **2a** with UV light triggered the formation of the addition product **3a** (Scheme 2), nitrone-sensitized photo-promoted process was envisaged as a background reaction. However, the acceleration of the transformation in the presence of 4,4′-dichlorobenzophenone led the authors to propose an alternative DCBP-sensitized photocatalytic sequence. Upon irradiation of DCBP, the triplet ^3^DCBP* was generated after intersystem crossing (ISC) from ^1^DCBP*. This gave rise to the formation of ketyl and α-aminoalkyl radicals **A** and **B**, through both PET and proton exchange events. Then, the α-aminoalkylation of **B** occured to nitrone **2a** to afford the aminoxyl radical intermediate **C**, providing, eventually, the addition product **3a** after a SET from ketyl **A** (recycling DCBP thereby) and proton exchange. The authors also assumed, in parallel, a radical chain process that would take place from the radical intermediate **C**, likely able to form the α-aminoalkyl radical **B** by hydrogen atom transfer (HAT), en route to the addition reaction of **B** to nitrone **1a** to construct **3a** via **C**.

**Scheme 3:**
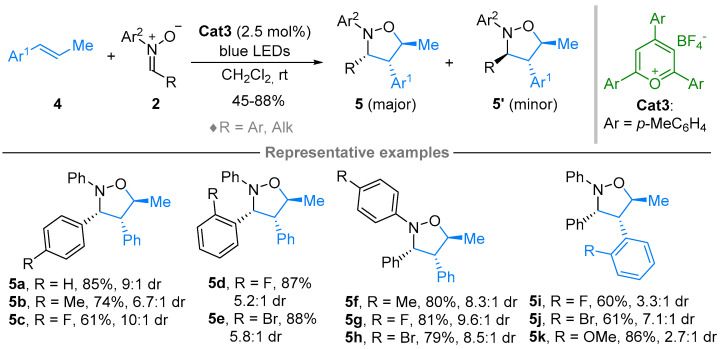
Photoredox-catalyzed formal (3 + 2) cycloaddition between β-methyl styrene derivatives and nitrones.

Following this pioneering contribution, Xia and colleagues published a formal (3 + 2) cycloaddition between β-methyl styrene derivative **4** and *N*-aryl nitrones **2** along with photoredox conditions thanks to a catalytic amount of pyrylium salt **Cat3** and blue LED irradiation (Scheme 3) [26]. Under these conditions, the major diastereoisomer **5** was obtained with aryl nitrones **5a**–**e** regardless of the substitution pattern, but lower yields and dr were observed with an alkyl nitrone. Various *N*-aryl nitrones **2** were tolerated to provide the corresponding products **5f**–**h** with moderate to good diastereoselectivities, but *N*-alkyl homologues were not tolerated under these conditions. Several functionalized β-methyl styrene derivatives **4** could be employed, to afford **5i**–**k** for instances, likewise for indene or unsubstituted styrene derivatives. In a complementary manner, the regioselectivity of this formal dipolar cycloaddition towards the formation of **5** was inverted with respect to the adducts obtained under classical thermal conditions. In a similar fashion, the authors demonstrated the addition reaction of indole [26].

**Scheme 4:**
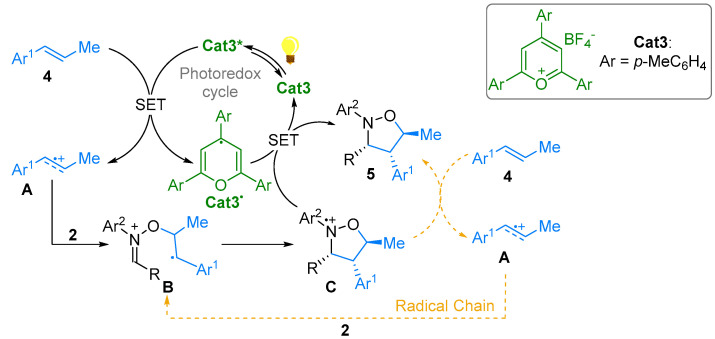
Proposed mechanism for the photoredox-catalyzed formal (3 + 2) cycloaddition between β-methyl styrene derivatives and nitrones.

As far as the mechanism is concerned, the excited Cat3* oxidized the alkene **4** into radical cation **A**, which underwent a regioselective addition of a nitrone **2** into intermediate **B** (Scheme 4) [26]. Then, a diastereoselective cyclization occurred and the obtained intermediate **C** was reduced into product **5** by transient **Cat3^●^** allowing the regeneration of **Cat3**. The fact that either (*E*)- or (*Z*)-β-methyl styrene precursors **4** led to similar diastereoselectivity was testified by a stepwise mechanism for the formal cycloaddition. On the other hand, a quantum yield measurement (Φ = 9) tended to show a radical chain process. Then, the authors proposed that radical cation **C** oxidized an alkene **4** into **A** allowing to initiate another catalytic cycle via radical **B**.

**Scheme 5:**
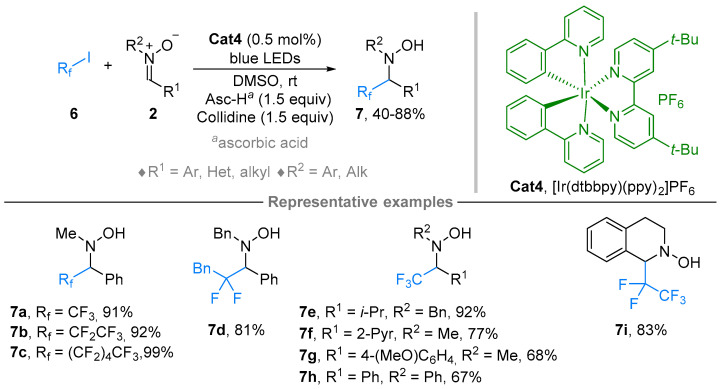
Photoredox-catalyzed reaction of fluoroalkyl iodide derivatives with nitrones.

In 2008, the group of Dilman extended the scope of radical addition to nitrones thanks to the versatility of photoredox catalysis (Scheme 5) [27]. Upon blue-LED irradiation, with a small amount of iridium complex **Cat4** (0.5 mol%), the addition of iodofluorinated compounds **6** was successfully performed to provide a wide array of hydroxylamines **7**. For instance, several types of fluorinated pendants (**7a**–**c**) could be introduced on either *N*-alkylated (**7d**–**g**) or *N*-arylated (**7h**) nitrone substrates all derived from aromatic or aliphatic aldehydes (R^1^). Even heterocycle-derived nitrones, such as an isoquinoline structure, were tolerated to form, e.g., tetrahydroquinoline **7i** in good yields. Only some limitations were encountered when highly hindered substrates were used, such as the one having a *tert*-butyl pendant or a disubstituted pattern, leading to decreased yields.

**Scheme 6:**
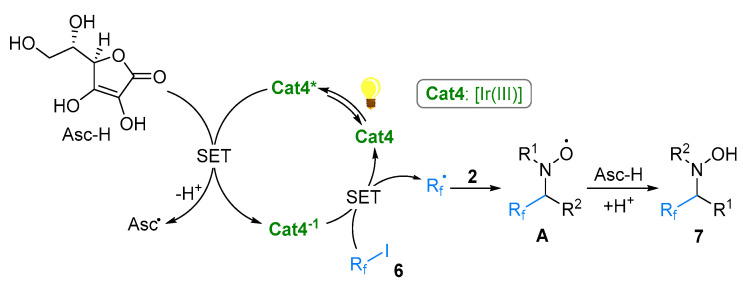
Proposed mechanism for the photoredox-catalyzed reaction of fluoroalkyl iodide derivatives with nitrones.

During mechanistic investigations, the authors showed that the presence of ascorbic acid as a reductant was key for the success of this sequence (Scheme 6) [27]. It was proposed, at first, as a reductive quenching of the excited photocatalyst Cat4* by ascorbic acid (likely its negative form Asc^−^) to provide the Ir(II) species **Cat4^−1^** (*E* = Ir(II)/Ir(III), −1.51 V) likely able to reduce the R_f_-I **6** (*E*_red_ = −1.22 to −1.77 V) into the corresponding radical R_f_^●^ (Scheme 6). Then, a radical addition reaction to nitrones **2** occurred to deliver the aminoxyl radical intermediate **A**, which can be reduced by the excess of Asc-H. Importantly, the reduction potential of −2 V estimated for nitrones **2** ruled out the first redox-reaction of these reagents.

**Scheme 7:**
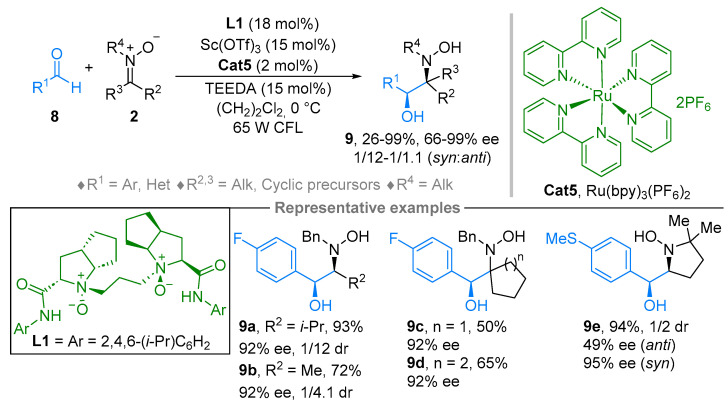
Photoredox-catalyzed reaction of aldehydes with nitrones.

Zhang, Lu and Huang and coworkers have made a breakthrough in 2009 by implementing the catalytic radical transformation of nitrones to the asymmetric synthesis level (Scheme 7) [28,29]. Motivated by the presence of the vicinal amino alcohol motif within a myriad of bioactive compounds, the authors demonstrated a photoredox-based pinacol type coupling between aromatic aldehydes **8** and nitrones **2** in the presence of Ru(bpy)_3_(PF_6_)_3_ **Cat5** (2 mol%) and the use of a 35 W compact fluorescent lamp. The corresponding coupling products **9** were obtained in good to excellent yields and good diastereoselectivity in most cases in favor of the *syn* isomer. Through a dual catalysis approach, it was shown that a catalytic amount of scandium Lewis acid together with the Feng’s chiral *N*,*N*′-dioxides ligand **L1** not only facilitate the C–C bond formation but also allows for a highly enantioselective process. This asymmetric sequence worked with various aromatic aldehydes **2** to afford either acyclic (**9a**–**b**) or cyclic (**9c**–**d**) amino alcohol compounds, even when the nitrone precursor was embedded within the cyclic framework (**9e**). The last case necessitated the use of *i*-Pr_2_EtN instead of TEEDA (*N*,*N*,*N*′,*N*′-tetraethylethylenediamine) as an amine and a slightly modified ligand to optimize the enantiomeric excess.

**Scheme 8:**
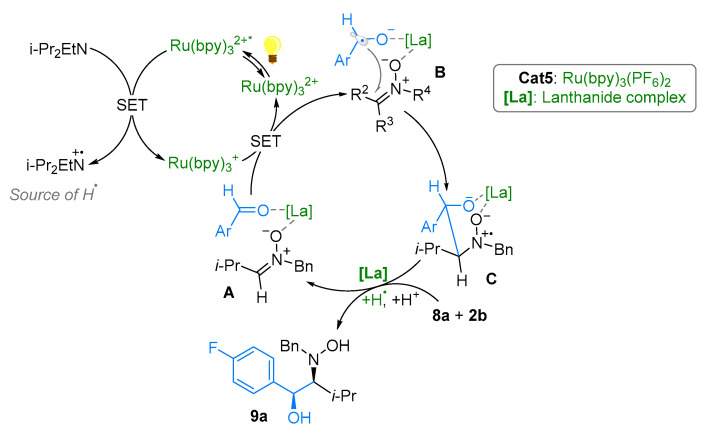
Proposed mechanism for the photoredox-catalyzed reaction of aldehydes with nitrones.

The radical nature of the mechanism was proven by both radical clock reactions using cyclopropyl ketone precursors **8** and an inhibition reaction observed in the presence of TEMPO [28,29]. Accordingly, it was assumed that an active Ru(bpy)_3_^+^ complex was formed after a reductive quenching of the corresponding Ru(bpy)_3_^2+^ by the Hünig base. The nitrone **2b** was engaged in a complex **A** between the Lewis acid (**La**) (lanthanide metal embedded with the chiral ligand **L1**) and the aldehyde **8a** (Scheme 8). This resulted in a Lewis acid activation of the aldehyde **8a** (half-wave potential changed from −1.86 V to −0.62 V vs. SCE (saturated calomel electrode) + Sc(OTf)_3_), which underwent a reductive SET to afford the ketyl radical intermediate **B**. Following a Zimmerman–Traxler-type transition state, supported by DFT calculation, a C–C bond construction took place to give rise to cation radical **C**, leading to the formation of the final product **9a** thanks to the hydrogen abstraction event from previously formed *i*-Pr_2_EtN^+^. Worthy of note, this reductive SET-triggered process upon photoredox conditions was complementary to the homologous samarium-based sequence, which usually started from the reduction of the nitrone **2** counterpart [28,29].

**Scheme 9:**
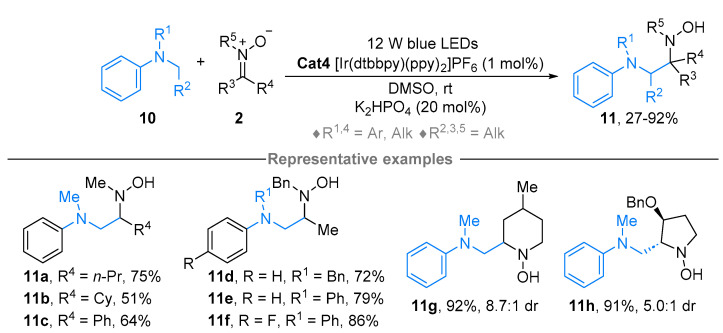
Photoredox-catalyzed reaction of *N*-aromatic α-aminoalkyl derivatives with nitrones.

As aforementioned [25], Itoh, Suga demonstrated the α-aminoalkylation reaction of nitrones **2** by using a UV-excited photosensitizer protocol (see Scheme 1). In 2019, the group of Xia and Pei-Qiang implemented this sequence while expanding the scope under visible light photoredox catalysis (Scheme 9) [30]. Under these safer and more efficient conditions, and in the presence of a catalytic amount of iridium complex **Cat4** (1 mol%) and K_2_HPO_4_ as a base in DMSO, the addition of amines **10** was observed and provided a smooth formation of diamines **11** in good-to-excellent yields. Under these conditions, the synthesis of various alkyl or aryl derived nitrones **11a**–**c** was achieved and various aniline derivatives were tolerated (**11d**–**f**). Cyclic nitrones could also be engaged in this transformation, leading to the corresponding hydroxylamines **11g** and **11h**, with a high diastereoisomeric ratio in the last case due to the steric influence of the Obn group at the α-position.

**Scheme 10:**
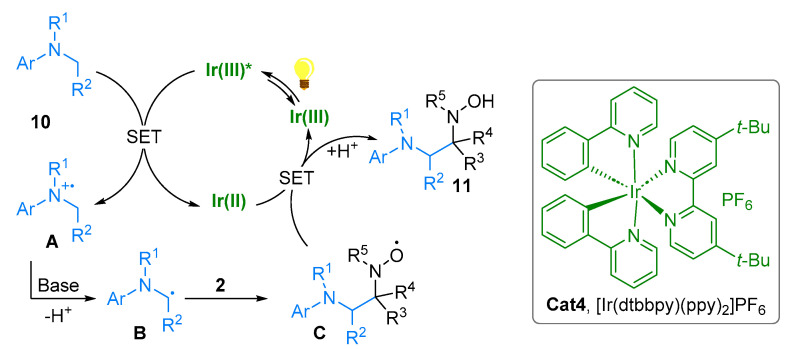
Proposed mechanism for the photoredox-catalyzed reaction of *N*-aromatic α-aminoalkyl derivatives with nitrones.

The authors proposed a catalytic pathway that commenced by the oxidation of the aniline **10** by the excited iridium(III)* complex Cat4* delivering, after deprotonation of the radical cation intermediate **A**, the radical species **B** (Scheme 10) [30]. The radical **B** added to nitrone **2** to furnish aminoxyl radical intermediate **C**, which eventually underwent a reductive SET by iridium (II) regenerating the initial catalyst Ir(III) and providing product **11** after protonation. On the other hand, the authors did not exclude that aminoxyl radical **C** could lead to a hydrogen abstraction of the radical cation **A**, also providing the reactive radical **B** after an Ir(II) reduction event.

**Scheme 11:**
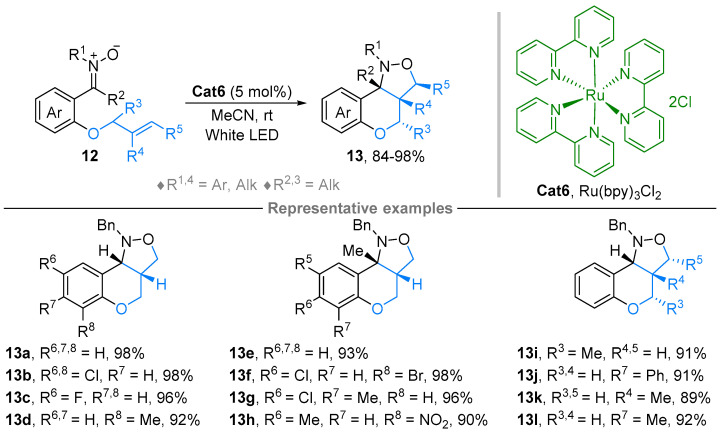
Photoredox-catalyzed intramolecular formal (3 + 2) cycloaddition.

The intermolecular formal (3 + 2) cycloaddition was demonstrated by Xia and colleagues upon photoredox catalysis in 2017 (see Scheme 3) [26]. Motivated by the elaboration of chromenoisoxazolidine derivatives, a core structure of bioactive compounds, Moura-Letts and coworkers tackled the intramolecular process, which required different conditions (Scheme 11) [31]. The authors showed that well-designed nitrone-alkene platforms **12** underwent an intramolecular dipolar cycloaddition reaction, giving rise to the formation of the tricyclic products **13** in high diastereo- and regioselectivity. This transformation required the use of 5 mol% of photocatalyst Ru(bpy)_3_Cl_2_ **Cat6** (a lower amount gave lower yields) in acetonitrile and a white LED as a source of light. Although the reaction could also be performed under thermic conditions, the photoredox approach proved to be more efficient (providing a cleaner reaction) and proceeded at room temperature. A large variety of chromenoisoxazolidine derivatives were thereby synthesized with various substitution pattern on the aromatic (**13a**–**d**) and the allylic (**13i**–**l**) moieties. Ketone-derived nitrones (R^2^ = Me) were also tolerated to allow the construction of products **13e**–**h**.

**Scheme 12:**
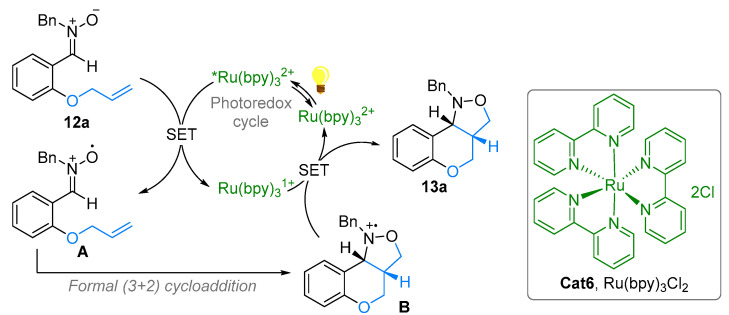
Proposed mechanism for the photoredox-catalyzed intramolecular formal (3 + 2) cycloaddition.

Concerning the mechanism, it was demonstrated that the sequence required light to proceed, which led the researchers to propose a redox-neutral catalytic cycle (Scheme 12) [31]. Upon white light irradiation, the photocatalyst Ru(bpy)_3_^2+^ (**Cat6**) was excited to *Ru(bpy)_3_^2+^ being able to oxidize the nitrone **12a** into the aminoxyl radical **A**, which triggered the formal (3 + 2) cycloaddition into **B**. Then, the thus-formed Ru(bpy)_3_^1+^ complex secured a reductive SET, recycling the Ru(bpy)_3_^2+^ photocatalyst and furnishing the product **13a**.

**Scheme 13:**
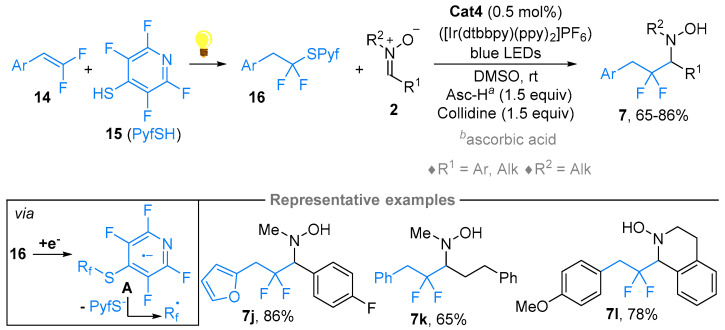
Photoredox-catalyzed reaction of *in situ*-generated difluoroalkyl derivatives with nitrones.

The group of Dilman developed the 4-tetrafluoropyridinylthio (PyfS) moiety, as a new redox active functional group for the generation of fluorinated radical (R_f_^●^) through the formation of the radical anion **A** (Scheme 13). The PyfS-functional group was easily installed onto difluorinated alkenes **14** making use of the thiol precursor **15** (PyfSH) under an electron proton transfer protocol to afford **16**. Thanks to the previously developed conditions for iodofluorinated compounds **6** in the presence of an iridium photocatalyst **Cat4** (see Scheme 5) [27], an efficient addition of PyfS-Rf **16** precursor to nitrones **2** occurred to provide a series of difluorinated hydroxylamines **7** (e.g., compounds **7j**–**l**). The photocatalytic mechanism is likely reminiscent to the one described with iodofluorinated compounds **6** (see Scheme 6). 

**Scheme 14:**
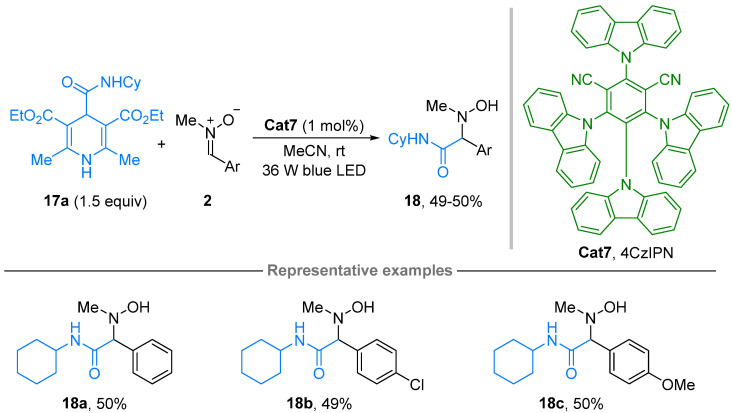
Photoredox-catalyzed reaction of 4-carbamoyl dihydropyridine derivatives with nitrones.

The group of Paixão recently developed a couple of SET strategies to perform the addition of radical species to dipoles such as azomethine imines (vide infra) and nitrones (Scheme 14) [32,33]. They used the dihydropyridine **17a**, which allowed the generation of a carbamoyl radical after the oxidative single electron transfer of the amine **17a** secured by the excited photocatalyst **Cat7** (4CzlPN) [33]. The authors proved the effectiveness of this carbamoylation protocol for the synthesis of three hydroxylamines **18a**–**c** in good yields. This interesting redox sequence was much more developed on azomethine imines dipoles as discussed in the next chapter [33].

**Scheme 15:**
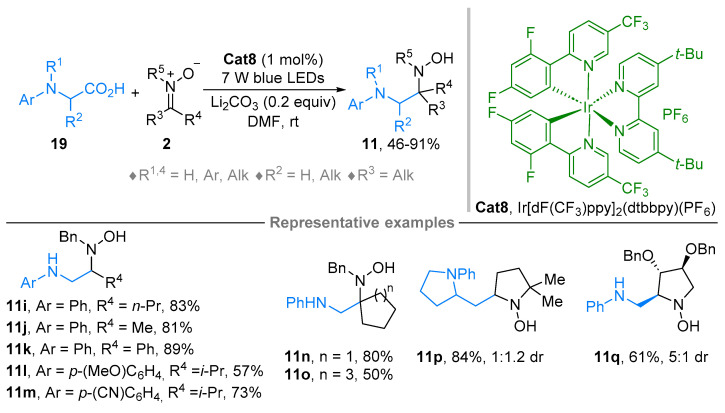
Photoredox-catalyzed reaction of α-amino acids with nitrones.

The oxidation decarboxylation sequence has emerged as a powerful strategy to generate alkyl radical species, a field in which naturally occurring and abundant α-amino acid derivatives proved to be versatile precursors [34]. In that vein, Huang and colleagues demonstrated that *N*-arylated α-amino acids **19** turned out to be able to add to nitrones **2** under blue LEDs irradiation in the presence of Ir[dF(CF_3_)ppy]_2_(dtbbpy)(PF_6_) **Cat8** (1 mol%) (Scheme 15) [35]. Importantly, lithium carbonate as a base was mandatory for getting high yield showing that a deprotonated carboxylic acid (carboxylate) was likely the precursor involved in this decarboxylative process. In these conditions, a smooth photoredox-catalyzed decarboxylation triggered an addition sequence eventually leading to the formation of hydroxylamines **11**. Various α-amino hydroxylamines having an alkyl or aryl pendant (**11i**–**m**) could be synthesized as well as products **11n**–**o** originated from the ketone nitrones. On the other side, for instance, cyclic nitrones could be engaged in this transformation giving pyrrolidine hydroxylamines **11p** and **11q**, with good diastereoselectivity in the last case.

**Scheme 16:**
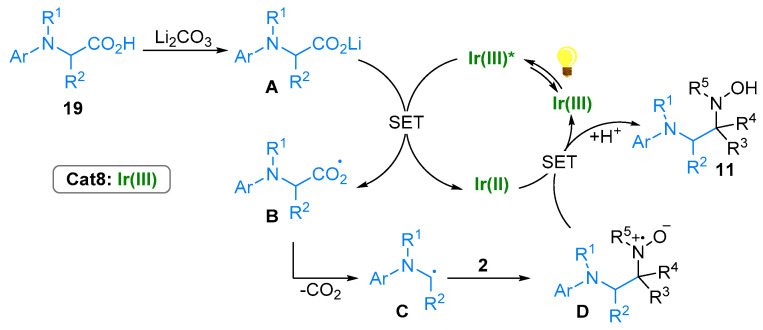
Proposed mechanism for the photoredox-catalyzed reaction of α-amino acids with nitrones.

It was proposed that the mechanism of this transformation stemmed from the formation of carboxylate **A** after deprotonation of the α-amino acid **19** (Scheme 16) [35]. An oxidative SET of this anion **A** by the excited iridium(III) photocatalyst **Cat8** allowed the formation of the radical **B** undergoing a decarboxylative event towards α-amino radical **C** thereafter. Then, an addition reaction took place to nitrone **2** to afford product **D**, which underwent a reduction to the final product **11** by Ir(II) complex and thereby securing a redox-neutral process.

### 2.2. Azomethine Imines

**Figure 4:**
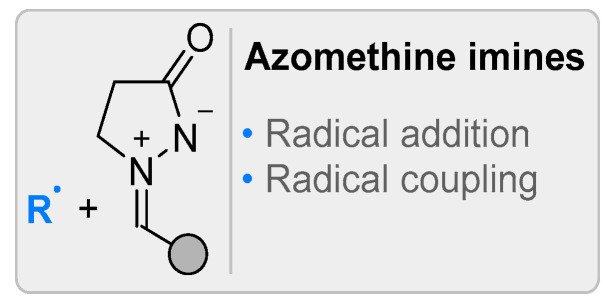
Radical-based transformations of azomethine imines.

The radical-based transformation of azomethine imines has only recently appeared in the literature by using essentially cyclic Dorn- and Otto-derived dipoles (Figure 4) [36]. This emerging synthetic approach complements the well-developed dipolar cycloadditions and nucleophilic additions of organometallic anionic reagents to these types of dipoles [37,38,39,40].

**Scheme 17:**
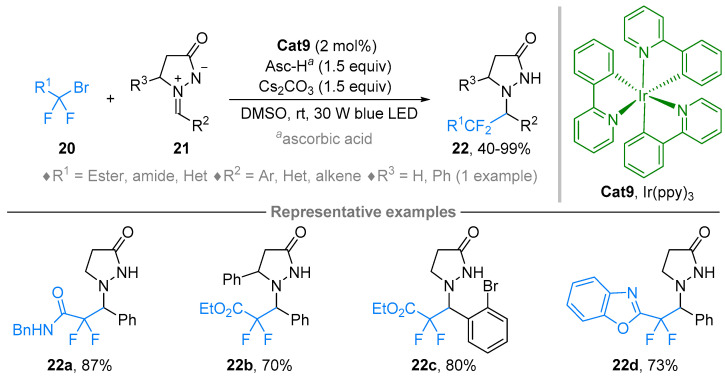
Photoredox-catalyzed reaction of *gem*-difluoroalkyl bromide derivatives with azomethine imines.

In 2019 (Scheme 17), the group of Yang, Chen, Xiang and collaborators demonstrated the formal addition of difluoroalkyl derivatives R^1^CF_2_Br **20** (R = CO_2_R, C(O)NR_2_, heterocycles) to azomethine imines **21** upon visible light photoredox catalysis. By means of a powerful reductant, namely the photocatalyst Ir(ppy)_3_ **Cat9** (*Ir(ppy)_3_, *E*_red_ = −1.82 V vs. Ag/AgCl in DMSO), in the presence of an excess of ascorbic acid (Asc-H) and Cs_2_CO_3_ as a base [41],various difluoroalkyl motifs (**22a**–**d**) flanked by ester, amide or heterocyclic moieties could be introduced.

**Scheme 18:**
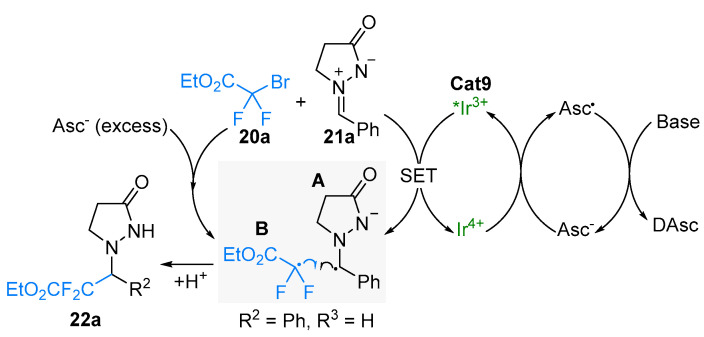
Proposed mechanism for the photoredox-catalyzed reaction of *gem*-difluoroalkyl bromide derivatives with azomethine imines.

A mechanistic investigation revealed that upon irradiation, the excited iridium(III) photocatalyst **Cat9** reduced the azomethine imine **21a** (*E*_red_ = −1.76 V vs. Ag/AgCl in DMSO) by a SET to form the corresponding radical intermediate **A** (Scheme 18). The radical **A** was subsequently engaged into a radical–radical coupling process with a difluoromethylene radical counterpart **B**, originated from the reduction in BrCF_2_CO_2_Et **20a** by an excess of ascorbate (Asc^−^), to provide product **22a**. The ascorbic acid (*E*_ox_ = +0.32 V vs. Ag/AgCl in DMSO) also regenerated the photocatalyst **Cat9** by reducing the Ir(IV) species (*E*_ox_ = +0.68 V vs. Ag/AgCl in DMSO) into Ir(III). The ascorbyl radical thus obtained disproportionated into both the dehydroascorbic acid (DAsc) and ascorbate (Asc^−^) in the presence of a base, the last species (Asc^−^) being useful for the reduction of **21a** into **B**.

**Scheme 19:**
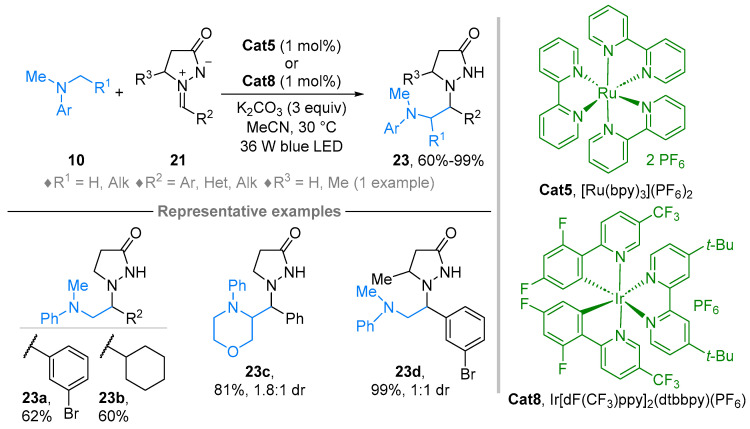
Photoredox-catalyzed reaction of *N*-aryl α-aminoalkyl derivatives with azomethine imines.

Shortly afterwards, the group of Paixão developed an efficient photocatalytic sequence allowing the α-amino alkylation of azomethine imines **21** into products **23** starting from a large array of *N*,*N-*dimethylaniline derivatives **10**, which required the use of a base such as K_2_CO_3_ and degassed conditions (Scheme 19) [32]. The corresponding pyrazolidinones **23** were obtained thanks to blue LED irradiation conditions either with ruthenium or iridium photocatalysts **Cat5** and **Cat8**, respectively. This strategy was advantageously applied to the late-stage modification of pharmaceutical ingredients. Next, stereoselective processes were also addressed, but moderate diastereoselectivities were observed. 

**Scheme 20:**
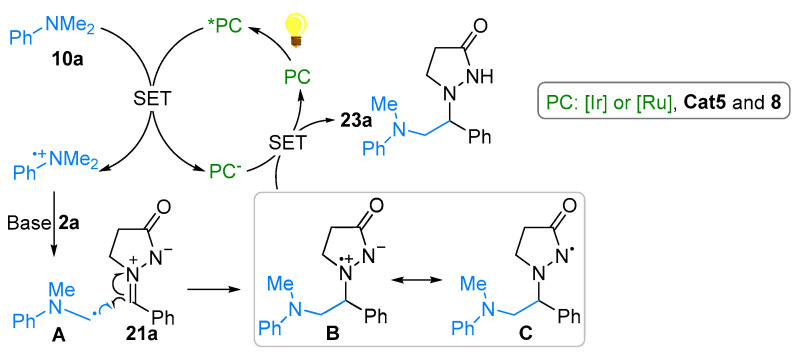
Proposed mechanism for the photoredox-catalyzed reaction of *N*-aryl α-aminoalkyl derivatives with azomethine imines.

The mechanistic investigation allowed the authors to propose a photocatalytic cycle by which the excited photocatalysts (*PC) **Cat5** (*E*_1/2_(Ru(II)*/Ru(I)) = +0.77 V vs. SCE in MeCN) or **Cat8** (*E*_1/2_(Ir(III)*/Ir(II)) = +1.21 V vs. SCE in MeCN) first oxidized the dimethylaniline **10a** (*E*_ox_ = 0.80 V vs. SCE in MeCN) leading to the radical **A** after deprotonation of the radical cation intermediate (Scheme 20) [32]. Then, an addition of the radical **A** took place to azomethine imine **21a** providing the radical cation **B**, or the amidyl intermediate **C** derived thereof, which underwent a reduction by the SET triggered by PC^−^ furnishing the desired product **23a** while regenerating the photocatalyst through an overall redox neutral sequence. The direct reduction of the azomethine imine **21a** (*E*_red_ = −1.66 V vs. SCE at 20 °C in MeCN) was ruled out based on the unsuitable reduction potential of **Cat5** and **Cat8** (*E*_red_ = −1.33 V vs. SCE and *E*_red_ = −1.37 V vs. SCE at 20 °C in MeCN). The Paixão’s tactic complements the one developed by Yang and colleagues [41], who claimed the first reduction event of the azomethine imine **21a** (Scheme 18 vs. Scheme 20).

**Scheme 21:**
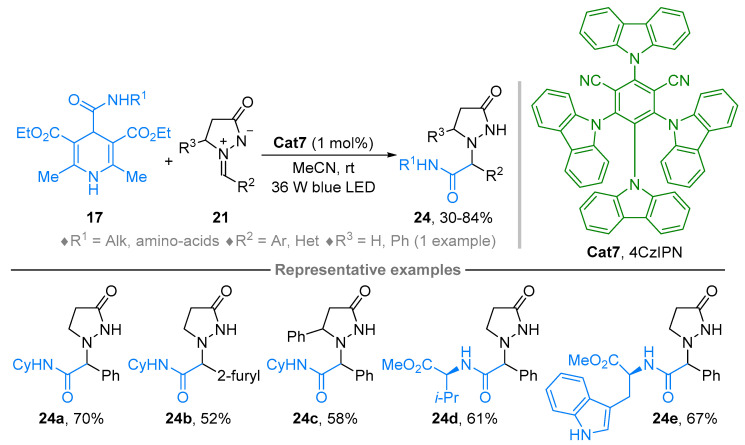
Photoredox-catalyzed reaction of 4-carbamoyl dihydropyridine derivatives with azomethine imines.

The Paixão group further developed this strategy by making use of readily available 1,4-dihydropyridines (DHPs) reagents **17** in order to install an amide moiety onto the azomethine imines **21** (Scheme 21) [33]. Thanks to the organic photocatalyst 4CzIPN **Cat7** and upon blue LED irradiation, a series of amide-derived pyrazolidinones **24** flanked by an aryl or heteroaryl moiety (R^2^) were synthesized. Importantly, these conditions were general enough for the synthesis of a large array of amino-acids (e.g., **24d**–**e**), peptides and steroid-like moieties (R^1^NH), which are important pendants in drug-like molecules.

**Scheme 22:**
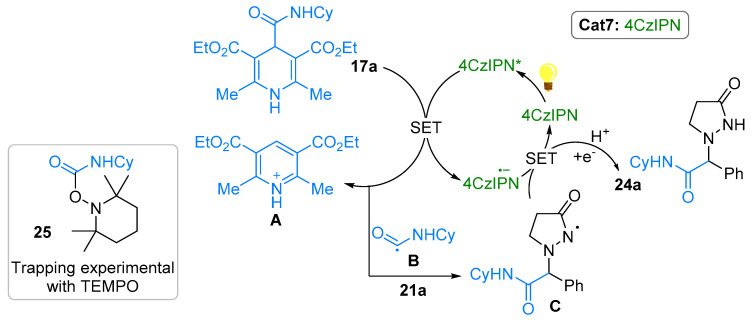
Proposed mechanism for the photoredox-catalyzed reaction of 4-carbamoyl dihydropyridine derivatives with azomethine imines.

In the spirit of the previous catalytic cycle (Scheme 20 vs. Scheme 22), the authors proposed a reductive quenching of the photocatalyst **Cat7** leading to an oxidation of the dihydropyridine **17a** (*E*_ox_ = +1.21 V vs. SCE) in the pyridinium cation intermediate upon SET, which released the protonated pyridinium **A** and carbamoyl radical **B [33]**. This sequence was secured by the triplet excited state of photocatalyst 4CzIPN **Cat7** (*E*_1/2_(PC*/PC^−^) = +1.35 V vs. SCE) and the presence of the radical species **B** was proven by a trapping experiment with TEMPO (Scheme 6). Then, the addition of the carbamoyl radical **B** to dipole **2a** occurred to form the supposed amidyl intermediate **C**, which underwent a reduction by 4CzIPN^●−^, followed by a protonation event towards the formation of product **24a**.

**Scheme 23:**
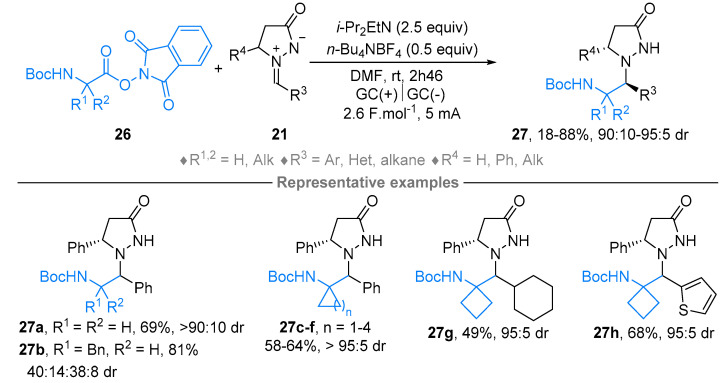
Photoredox-catalyzed reaction NHPI redox-active esters with azomethine imines.

In these previous pioneering and insightful contributions, upon photoredox catalysis, the use of chiral azomethine imine precursors **21** was mentioned but sparingly exploited in radical processes and led to modest or undescribed diastereoselective ratios. Oudeyer, Brière and coworkers addressed this issue by using an alternative electrochemical approach (Scheme 23) [42] under convenient galvanostatic conditions with glassy carbon electrodes in an undivided cell. In the presence of Hünig base, the redox-active esters (RAEs) *N*-(acyloxy)phthalimides (NHPs) **26** were added diastereoselectively to dipoles **21** (90:10 to >95:5 dr) to furnish, after purification on silica gel column chromatography, a single diastereoisomer of pyrazolidinones **27**. Primary, secondary and tertiary radical intermediates were tolerated (e.g., providing **27a**–**c**), and improved stereoselectivity were obtained with the more sterically hindered precursors. Azomethine imines with an heteroaromatic (**27h**) or aliphatic (**27g**) were compatible with these conditions. Importantly, the use of an enantioenriched azomethine imine (98% ee) showed no erosion of the enantioselectivity during this radical process. 

**Scheme 24:**
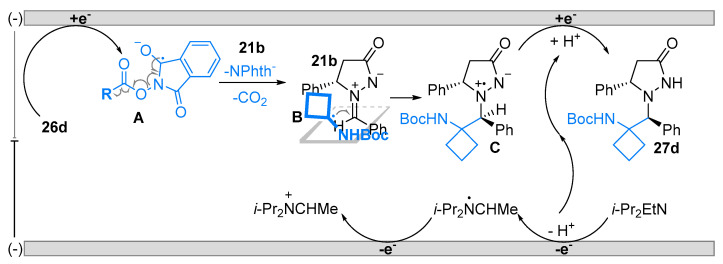
Proposed mechanism for the photoredox-catalyzed reaction NHPI redox-active esters with azomethine imines.

At that stage, it was believed that a first cathodic reductive SET generated the radical intermediate **A** from the cyclobutene derived RAE **26d** to afford, after the fragmentation-decarboxylation events, radical **B** (Scheme 24). An addition of **B** to the less hindered face of the (*Z*)-azomethine imine **21b** provided the cation radical **C**, which was sequentially reduced at the cathode leading to product **27d** after protonation. The cyclic voltammetry investigation demonstrated the ability of the RAE **26d** (*E* = −1.27 V vs. SCE in *N*,*N*-dimethylformamide—DMF) to be reduced before the azomethine imine **21b** (*E* = −1.66 V vs. SCE in DMF), while the Hünig base (*E* = +0.88 V vs. SCE in DMF) served as a sacrificial reducing agent during the counter reaction and a source of proton. It has been proposed that this amine was oxidized first in galvanostatic conditions and allowed, thereby, a protection of the formed product **27d**, which highlighted two oxidation waves at +1.12 V and +1.56 V, respectively.

### 2.3. Various Dipoles

**Scheme 25:**
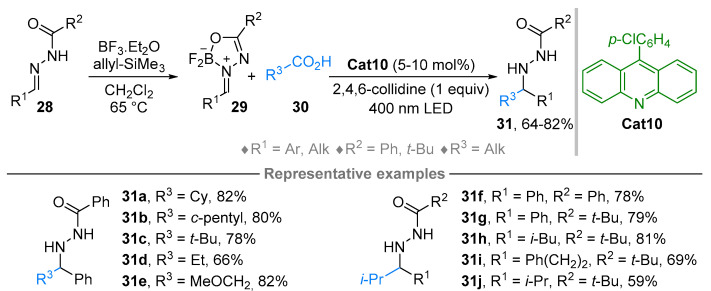
Photoredox-catalyzed reaction of carboxylic acid derivatives to *N*-acylhydrazones.

Among imine derivatives, hydrazones are known to be versatile platform molecules for the addition of radical species and serve as model substrates to investigate these processes [12,13,14]. Although the activation of hydrazones by Lewis acids, such as BF_3_.Et_2_O, is known to favor the radical addition reaction, Dilman and coworkers reported in 2021 an insightful investigation on boron chelate intermediates [43] (Scheme 25). Indeed, in the presence of boron trifluoride diethyl etherate, the *N*-acylhydrazones **28** were transformed into the corresponding difluoroboryl complex **29**, which can be isolated or used in situ for the subsequent radical addition reaction. Formally, this type of structure **29** is reminiscent of the aforementioned dipole species and hereby discussed for comparison purposes. The presence of the well-suited acridine photocatalyst **Cat10**, upon irradiation (400 nm, 60 W LED), triggered a decarboxylative event of carboxylic acid **30** allowing a radical addition to dipole-like **29** leading to the addition products **31** in good yields. For instance, *N*-benzoyl hydrazones (R^2^ = Ph) underwent an addition reaction of primary, secondary and tertiary radicals to give products **31a-31e**, and *N*-pivalic hydrazones (R^2^ = *t*-Bu) were also reactive in these conditions to provide products **31f**–**j**. In this case, a protocol with an in situ formation of the dipole-like species **29** was successfully used. Importantly, several hydrazones **28** (precursors of **29**) were evaluated but did not show any expected product formation.

**Scheme 26:**
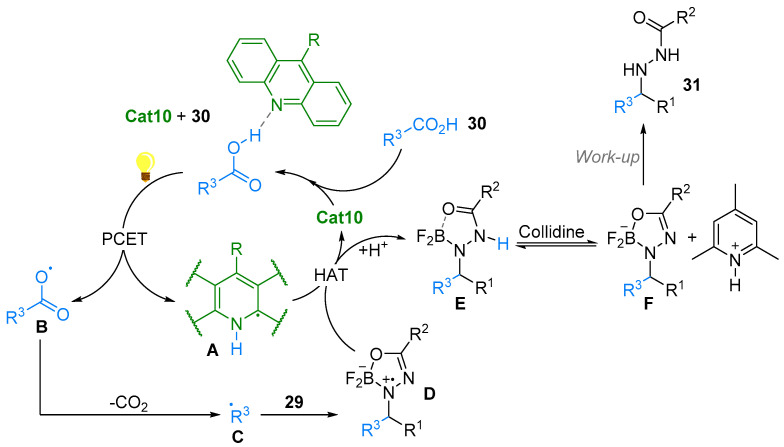
Proposed mechanism for the photoredox-catalyzed reaction of carboxylic acid derivatives to *N*-acylhydrazones.

Regarding the mechanism (Scheme 26), it was proposed that a hydrogen bonding occurred between the carboxylic acid partner **30** and acridine-derived photocatalyst **Cat10 [43]**. Upon irradiation a light-induced proton-coupled electron transfer (PCET) took place to furnish radicals **B** and, subsequently, **C** after decarboxylation. Then, an addition to iminium **29** occurred, giving rise to the supposed stabilized radical five-membered ring species **D**. Eventually, either a hydrogen atom transfer (HAT), or a SET followed by a protonation sequence, occurred and led to chelate product **E**, as a precursor of the expected final product after workup. The 2,4,6-collidine base was supposed to drive the protonation equilibrium toward **F**, preventing thereby a parasitic proton transfer event from intermediate **E**.

**Scheme 27:**
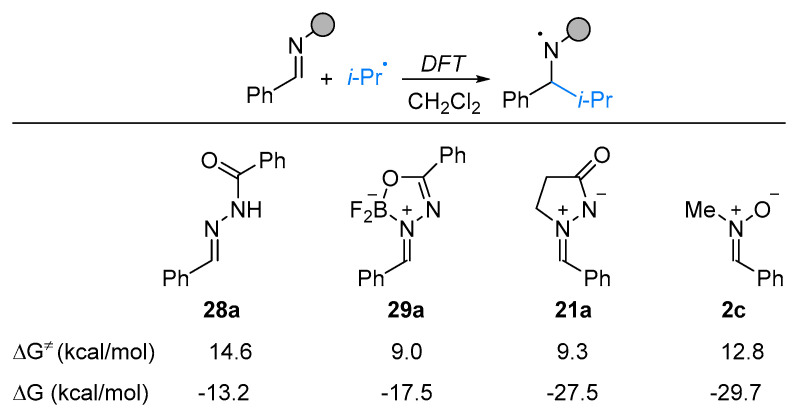
DFT calculations for the addition of *iso*-propyl radical to various C-N double bond derivatives including dipoles.

Interestingly, the authors performed DFT calculations on various imine derivatives with the addition of *iso*-propyl radical as a model reaction (Scheme 27). The addition of the *iso*-propyl radical was more facile with the difluoroboryl complex **29a** in comparison to the hydrazone **28a**. Interestingly, in spite of the addition reaction of dipoles such as nitrone **2c** or azomethine imine **21a** being much more exergonic, the radical process was faster (transition state lower in energy) with iminium **29a**, especially with regard to nitrone **2c**. The authors assumed this phenomenon to be due to the radical stabilization within the boron–chelate structure.

### 2.4. Pyridinium-N-Oxide

**Figure 5:**
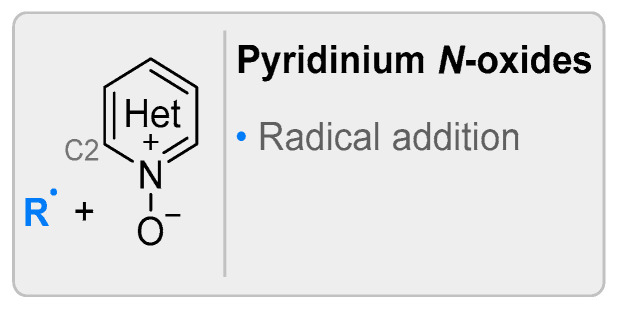
Radical-based transformations of pyridinium *N*-oxides.

While nucleophilic addition to arene *N*-oxides has been known since the beginning of the 20th century, Minisci-type radical addition only emerged a decade ago [22,23]. Since then, and thanks to the development of visible-light redox processes, *N*-oxides have become a great platform for C–H functionalization of heterocycles, as further exposed (Figure 5). It is worth mentioning that, in contrast to the classical Minisci reaction from heterocycles, starting with pyridine *N*-oxides provides an almost exclusive regioselectivity towards the formation of the C2-substituted product, explaining the wide interest that these moieties have raised. This regioselectivity was postulated to arise from electrostatic interactions between the pyridinium *N*-oxide and the radical precursor [44].

**Scheme 28:**
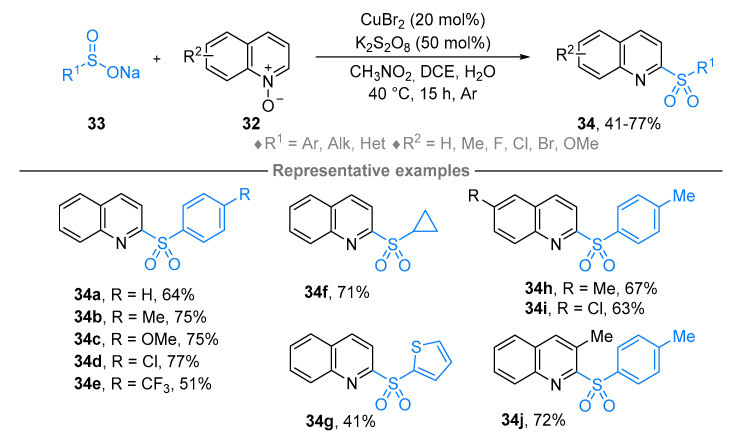
Cu(II)-catalyzed reaction of sodium sulfinate with quinoline *N*-oxides.

In 2016, Han, Pan and coworkers reported the deoxygenative C2-sulfonylation reaction of quinoline *N*-oxides **32** in the presence of sodium sulfinate **33** and potassium persulfate as the radical initiator and catalyzed by copper(II) bromide (Scheme 28) [45]. Variation in the substituents on the sulfinate and quinoline moieties were carried out with aryl (**34a**–**e**), alkyl (**34f**) and heteroaryl (**34g**) groups tolerated for the first one and methyl and halogens groups (**34h**–**j**) for the latter ones. Remarkably, exclusive formation of C2-functionnalized products was observed, regardless of the substitution pattern on the azacycles **32**.

**Scheme 29:**
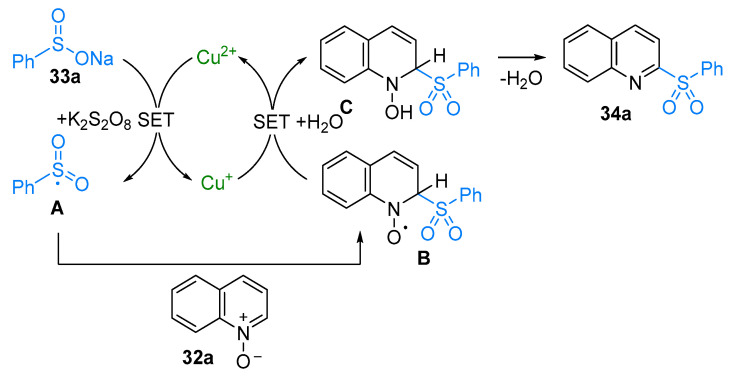
Proposed mechanism for the Cu(II)-catalyzed reaction of sodium sulfinate with quinoline *N*-oxides.

The reaction was believed to start with the oxidation of the sulfinate radical **33a** by Cu(II) or K_2_S_2_O_8_ (Scheme 29). Intermediate **A** reacted with the quinoline *N*-oxide through a Minisci-like pathway to deliver intermediate **B**, which was further reduced in the presence of water to deliver hydroxylamine intermediate **C**. The desired product **34a** was obtained by elimination of water from intermediate **C**.

**Scheme 30:**
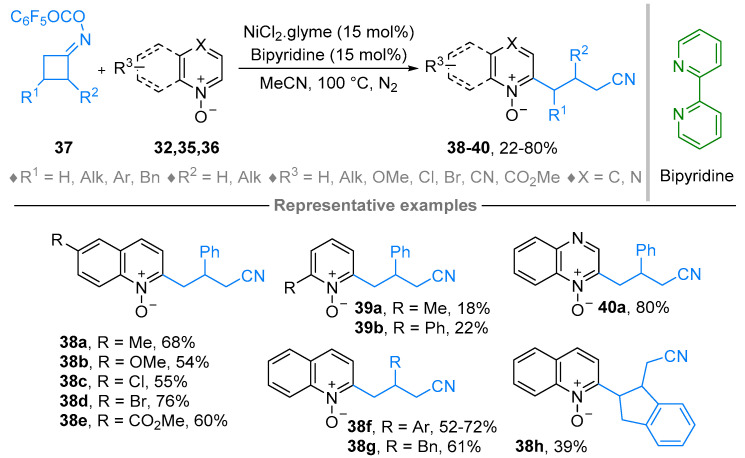
Ni(II)-catalyzed cyanoalkylation of azaarene *N*-oxides.

The group of Guo developed the catalytic cyanoalkylation of heteroaromatic *N*-oxides **32** (quinoline), **35** (pyridine) and **36** (quinoxaline) via C–C bond cleavage of cyclobutanone oximes **35** (Scheme 30) [46]. NiCl_2_.glyme surrounded by bipyridine ligands proved to be the best catalyst for this reaction, which still required a heating of 100 °C. Nevertheless, a broad scope was obtained, as quinolines (**38a**–**h**), isoquinolines, pyridines (**39a,b**), quinazoline, quinoxaline (**40a**) and benzoquinoline *N*-oxides were successfully applied to this methodology. It is worth mentioning that substitution was tolerated at any position on the quinoline. Moreover, cyclobutanone oximes bearing aryl, benzyl or alkyl groups on the C3 position (**38f,g**), as well as 3,3-disubstituted and tricyclic oximes (**38h**) were efficiently converted to the corresponding products. 

**Scheme 31:**
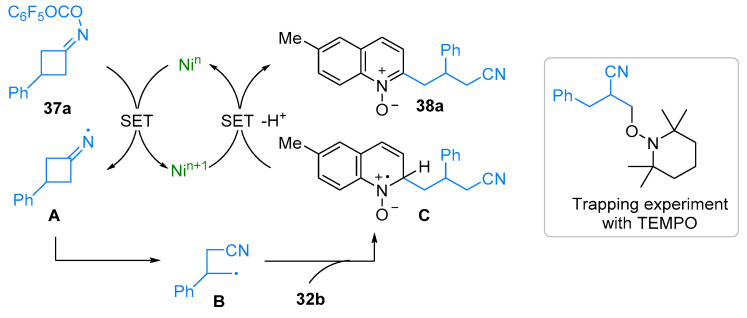
Proposed mechanism for the Ni(II)-catalyzed cyanoalkylation of quinoline *N*-oxides.

The mechanism proposed by the authors started with a SET from nickel to cyclobutanone oxime **37a** (Scheme 31). The formed radical intermediate **A** underwent a C–C bond cleavage via *β*-carbon elimination to generate **B**, which added to the quinoline *N*-oxide **32b**. The radical cation **C** was then oxidized by nickel and rearomatized by loss of a proton to release product **38a** and regenerated the catalyst. The presence of radical intermediate **B** was confirmed by trapping experiments with TEMPO and the entire mechanism was supported by radical clock trials, as well as intermolecular competitive and parallel experiments.

**Scheme 32:**
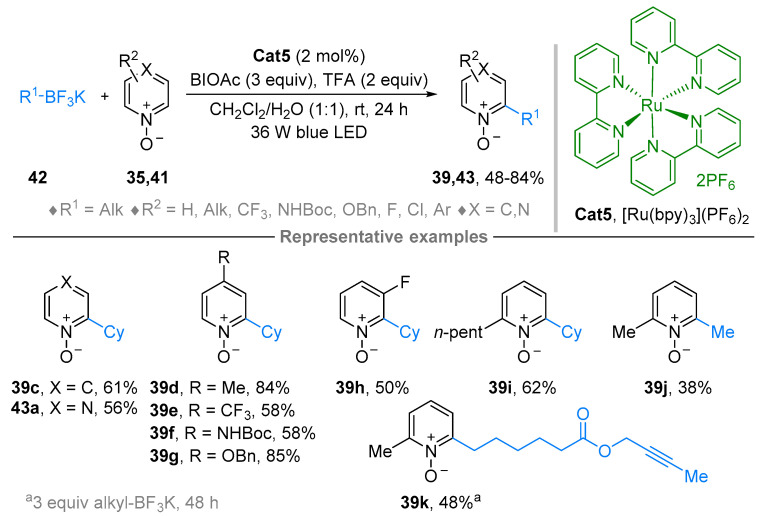
Photoredox-catalyzed reaction of azaraene *N*-oxides with potassium alkyl trifluoroborates.

Shortly afterwards, Xu and coworkers elaborated a new method to introduce alkyl groups at the C2 position of pyridine **35** and pyrazines **41** *N*-oxides using potassium alkyl trifluoroborates **42** and visible light photoredox catalysis to generate the alkyl radical of interest (Scheme 32) [47]. This methodology was applied to pyridine (**39c**) and pyrazine *N*-oxides (**43a**). The former heterocycle was functionalized at any position, by electron-donating or -withdrawing groups without affecting the regioselectivity of the reaction (**39d**–**k**). Concerning the scope of the potassium alkyl trifluoroborates, primary and secondary alkyl chains, containing functional groups such as esters, alkynes, benzyls or halogens, were successfully introduced (**39i**–**j**).

**Scheme 33:**
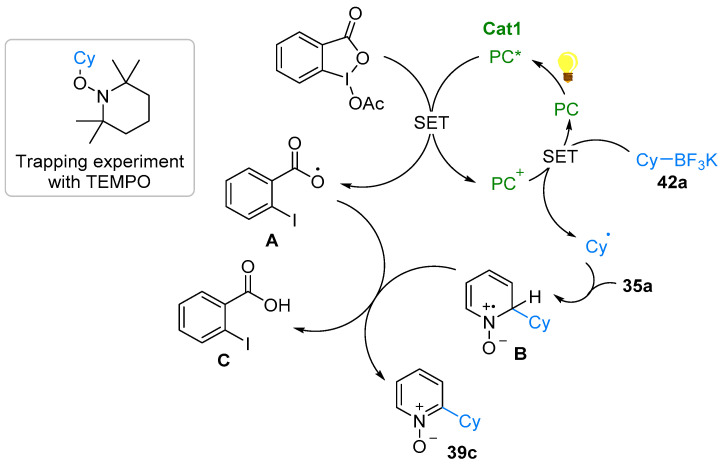
Mechanism for the photoredox-catalyzed reaction of azaraene *N*-oxides with potassium alkyl trifluoroborates.

Based on the experimental results and previous reports, the authors proposed an oxidative quenching cycle, reducing 1-acetoxy-1,2-benziodoxol-3-(1H)-one (BIOAc) into the corresponding alkoxy radical **A** (Scheme 33). The photocatalyst was regenerated by single-electron transfer from the alkyl trifluoroborate **42a** to form the alkyl radical, which further reacted with the pyridine *N*-oxides **35a**. The formation of alkyl radicals was confirmed by trapping experiments with TEMPO and BHT, the corresponding alkyl-TEMPO adduct being isolated. Hydrogen atom transfer from **B** yielded the desired product **39c** and 2-iodobenzoic acid **C**. Intermolecular (*k*_H_/*k*_D_ = 1.1) and intramolecular (*k*_H_/*k*_D_ = 1.2) kinetic isotope effects (KIE) were found to be almost equal to 1, ruling out the C–H bond cleavage being the rate-determining step.

**Scheme 34:**
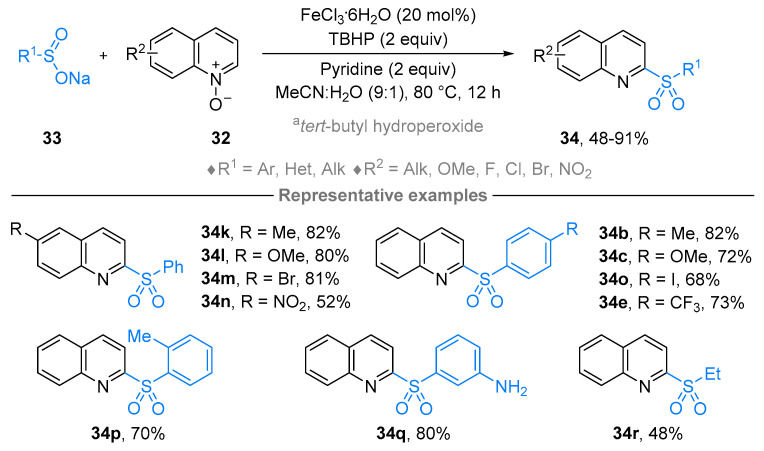
Fe(III)-catalyzed reaction of sodium sulfinates with quinoline *N*-oxides.

Inspired by the article from Han and Pan, An and coworkers developed a similar reaction catalyzed by iron(III) trichloride hexahydrate (Scheme 34) [48]. Their scope was comparable to the previous report, with electron-donating and -withdrawing groups tolerated at the C6 (**34k**–**n**), C3 and C4 position of the quinoline *N*-oxides. Aromatic sulfinates (**34b,c,e,o,p**) were mostly engaged in this reaction, even though examples of heteroaromatic and one example of alkyl sulfinate (**34r**) can be found. The mechanism is similar to the one proposed in Scheme 29.

**Scheme 35:**
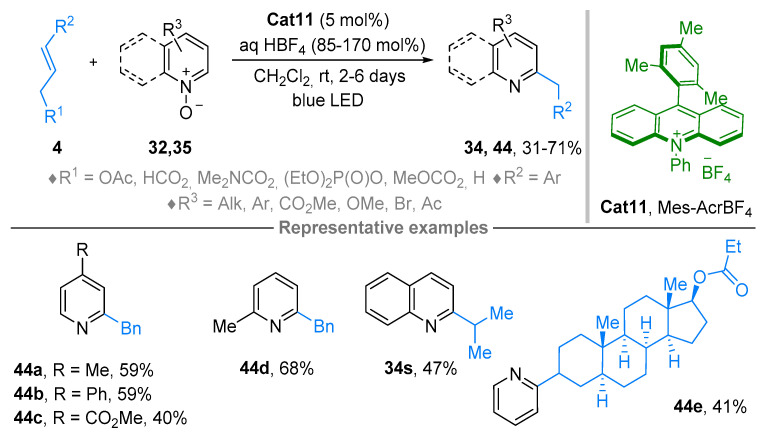
Photoredox-catalyzed reaction of quinoline *N*-oxides with electron-rich alkenes.

In 2018, Murakami, Miura and coworkers extended the existing methods for *ortho*-alkylation of pyridine **35** and quinoline **32** *N*-oxides to electron-rich alkenes **4** by means of visible light photocatalysis (Scheme 35) [49]. The organic photocatalyst Mes-Acr^+^BF_4_^−^ delivered the best results in acidic media at room temperature. The mild conditions enabled the functionalization of pyridine (**44a**–**d**) and quinoline (**34s**) *N*-oxides, bearing electron-donating (**44a,b**) or -withdrawing groups (**44c**). While the regioselectivity was high when the heterocycle was substituted at the C2 (**44d**) and C4 (**44a**–**c**) position, it was poor when substituted at the C3 position. Importantly, this method was applied to more complex molecules (**44e**) to prove its potential as a late-stage functionalization protocol.

**Scheme 36:**
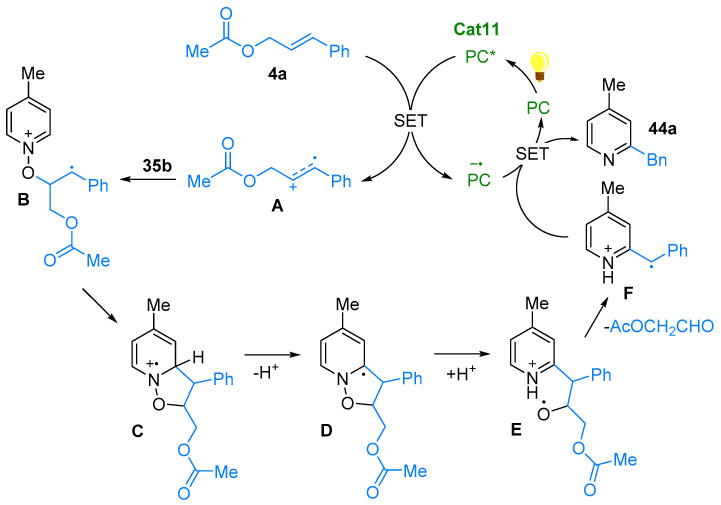
Proposed mechanism for the photoredox-catalyzed reaction of quinoline *N*-oxides with electron-rich alkenes.

The authors assumed that the mechanism proceeded via a reductive quenching cycle, in which the excited photocatalyst, oxidized the alkene **4a** to form the radical cation **A** (Scheme 36). Pyridine *N*-oxide **35b** reacted with **A** at the negatively charged oxygen center to give the cyclized radical intermediate **C**, which formed the C-centered radical **D** by loss of a proton. Intermediate **D** underwent a β-N–O bond scission driven by aromatization and promoted by an acidic media to deliver the O-centered radical intermediate **E**. The subsequent β-C–C bond scission released the C-centered radical intermediate **F** by loss of an aldehyde fragment. The catalytic cycle was closed by reduction of **F** to deliver the final product **44a** and regenerate the photocatalyst.

**Scheme 37:**
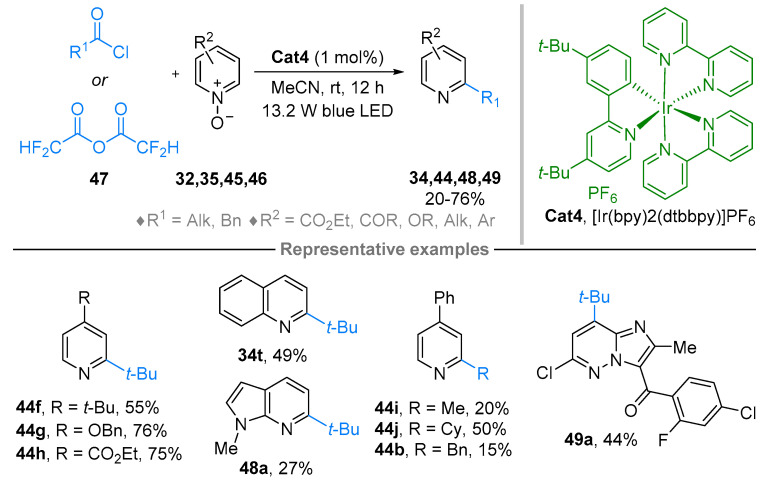
Photoredox-catalyzed reaction of azaarene *N*-oxides with acyl chlorides or difluoroacetic anhydride.

In line with the abovementioned report, the group of Stephenson described a mild method to perform *ortho*-alkylation of heterocycle *N*-oxides **32, 35, 45, 46** by the reductive generation of alkyl radicals under photoredox conditions (Scheme 37) [50]. The radical intermediates were obtained from acyl chlorides or difluoroacetic anhydride **47**. Even though the yields were generally moderate, the scope was wide, with good functional group tolerance, as underlined by the substituents on the heteroarenes (**44f**–**h**), and mostly the alkylation of biologically relevant molecules (**49a**). The reaction can also be performed on pyridine, quinoline (**34t**) and azaindole (**48a**). The mechanism of the reaction was not disclosed by the authors.

**Scheme 38:**
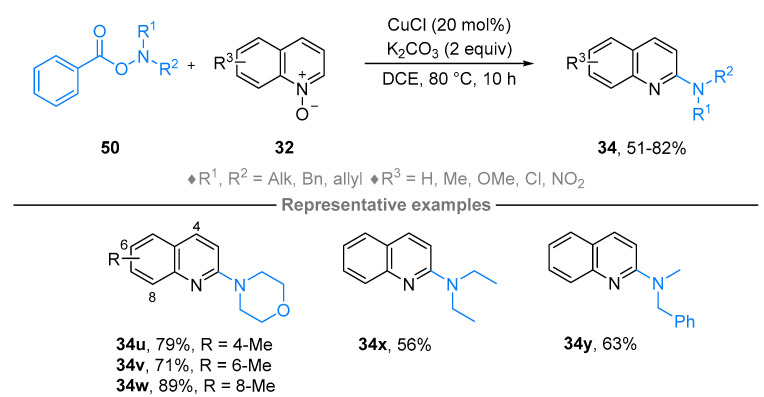
Cu(I)-catalyzed reaction of quinoline *N*-oxides with *O*-benzoylhydroxylamines.

At the same time, Wang and coworkers reported the C2-amination of quinoline *N*-oxides **32** (Scheme 38) [51]. They took advantage of *O*-benzoylhydroxylamines **50** as radical precursors, which enabled, in combination with 1,2-dichloroethane (DCE), the delivery of the corresponding quinoline **34** without any other reducing reagents. Substitution on the quinoline did not affect the yield (**34u**–**w**). Cyclic (**34u**–**w**) and acyclic symmetrical (**34x**) and asymmetrical (**34y**) amines were also successfully introduced.

**Scheme 39:**
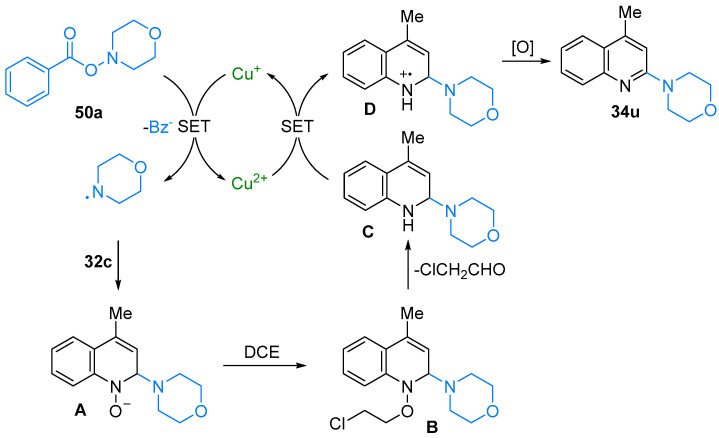
Proposed mechanism for the Cu(I)-catalyzed reaction of quinoline *N*-oxides with *O*-benzoylhydroxylamines.

The mechanism suggested by the authors began with the reduction of the *O*-benzoyl hydroxylamine **50a** to form the morpholine radical, which added to the quinoline *N*-oxide **32c** to deliver **A** (Scheme 39). A double reduction was required to obtain **A**, but this step was not further commented by the authors. Intermediate **A** reacted then with DCE, and the resulting intermediate **B** lost the chlorinated aldehyde via a N–O cleavage to deliver intermediate **C**. The latter was oxidized by the copper catalyst via single electron transfer and was further oxidized by oxygen or quinoline *N*-oxide **32c** to release the desired product **34u**. 

**Scheme 40:**
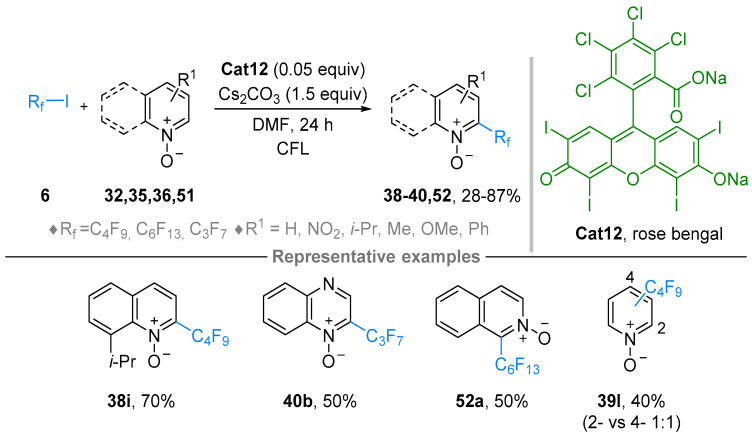
Photoredox-catalyzed reaction of azaarene *N*-oxides with perfluoroalkyl iodides.

Shortly afterwards, the group of Postigo described the photocatalyzed perfluoroalkylation of heteroarenes *N*-oxides **32, 35, 36, 51** using rose Bengal as the photocatalyst and iodoperfluoroalkyl reagent **6** (Scheme 40) [52]. While a wide diversity of heteroarenes was successfully utilized in this reaction (**38i**, **39l**, **40**, **52a**), the regioselectivity remained low for pyridine derivatives (**39l**).

**Scheme 41:**
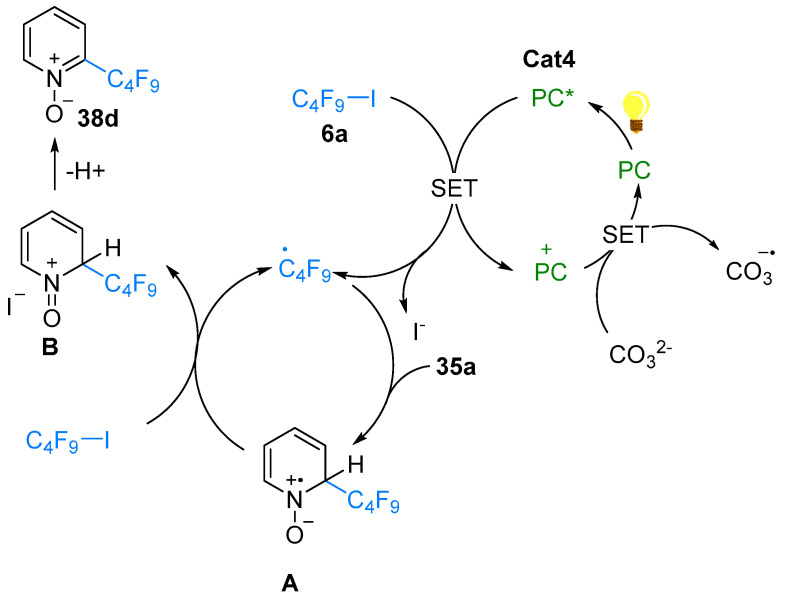
Proposed mechanism for the photoredox-catalyzed reaction of azaarene *N*-oxides with perfluoroalkyl iodides.

The proposed mechanism was initiated by the excitation of the photocatalyst, which reduced the perfluoroiodo reagent **6a** (Scheme 41). The perfluorinated radical added to the pyridine *N*-oxide **35a** to deliver the radical cation **A**. This intermediate reacted with the perfluoroiodo reagent to deliver **B** and regenerated the perfluoroalkyl radical. After a rearomatization process by loss of a proton, **38d** was obtained. To close the photocatalytic cycle, the catalyst was reduced by the carbonate anion.

**Scheme 42:**
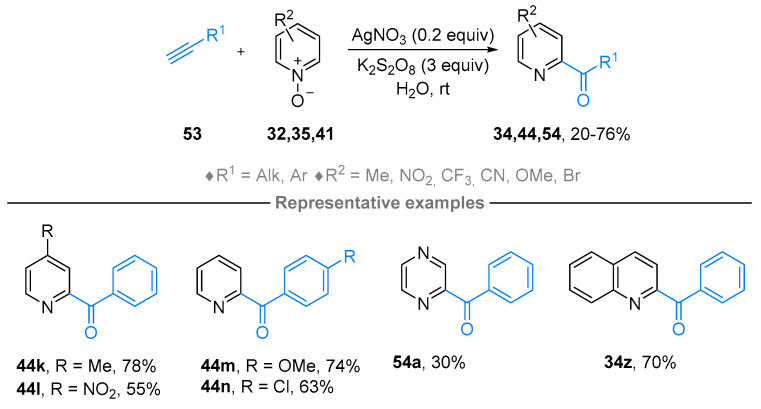
Ag(I)-catalyzed reaction of azaarene *N*-oxides with alkynes.

Later on, the group of Singh reported the *ortho*-acylation of heteroarene *N*-oxides **32, 35, 41** with alkynes **53** (Scheme 42) [53]. The combination of silver nitrate and potassium persulfate permitted the formation of an electrophilic *N*-oxide radical, which reacted with electron-rich alkynes **53**. The pyridine *N*-oxides **35** were substituted by electron-donating and -withdrawing groups (**44k,l**), such as the aromatic on the alkyne (**44m,n**). Other functionalized heteroarenes, such as pyrazine (**54a**) and quinoline (**34z**) were also successfully obtained under these reaction conditions. 

**Scheme 43:**
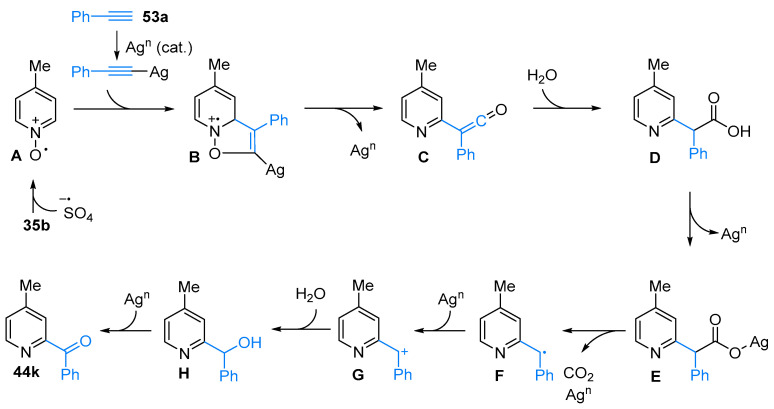
Proposed mechanism for the Ag(I)-catalyzed reaction of azaarene *N*-oxides with alkynes.

The mechanism described for this reaction is rather complex and required the involvement of several transfers of electrons, performed by species resulting from the reaction between silver nitrate and potassium persulfate (Scheme 43). Substrate **32a** was oxidized by a persulfate radical anion to form intermediate **A**, which reacted then with in situ generated silver acetylide to yield the radical cation **B**. This intermediate rearranged further into the ketene compound **C**. Addition of water to **C** resulted into the more stable carboxylic acid **D**. Intermediate **D** further reacted with silver to deliver the carboxylate **E**, which released CO_2_ to yield the radical compound **F**. Intermediate **F** was then oxidized and reacted with water to form **H**, further oxidized into the desired product **44l**. Intermediates **B**, **C** and **E** were confirmed by LC-MS experiment.

**Scheme 44:**
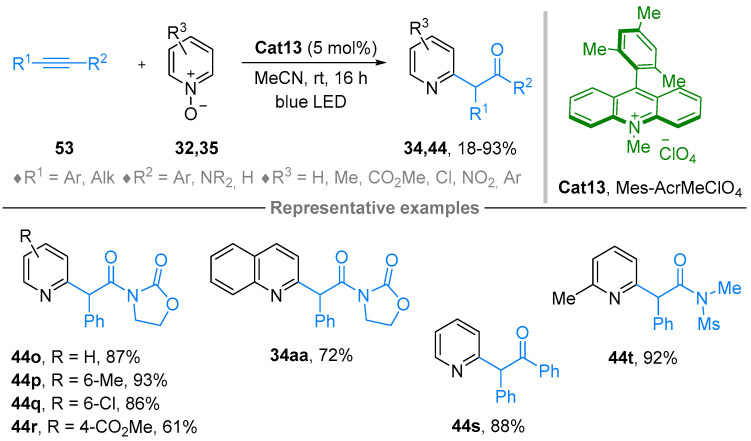
Photoredox-catalyzed alkylation of azaarene *N*-oxides with alkynes.

In line with the previous work by Murakami and coworkers with alkene, the group of Deng applied the method to alkynes **53**, which provided access to α-(2-pyridinyl) benzyl amides/ketones **44** by means of photoredox catalysis (Scheme 44) [54]. Substitution on the pyridine *N*-oxides **35** did not show a significant impact on the yield (**44o**–**s**) and quinoline *N*-oxides **32** could also be subjected to the reaction conditions (**34aa**). Alkynes functionalized by arenes or amines were also successful partners in this reaction (**44s,t**). The mechanism resembles the one from Scheme 36 and thus will not be further described.

**Scheme 45:**
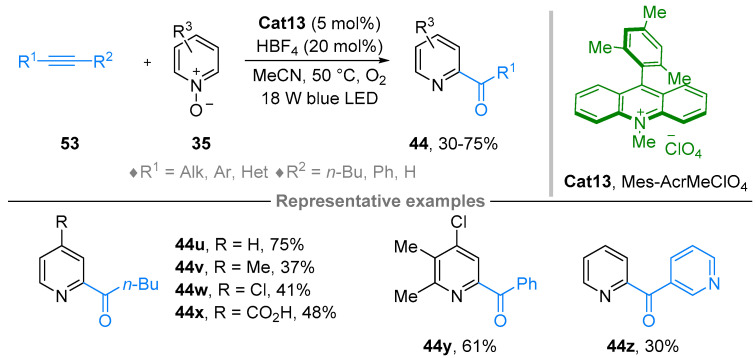
Photoredox-catalyzed acylation of pyridine *N*-oxides with alkynes.

Shortly afterwards, the group of Wu extended the scope of the abovementioned reaction and disclosed the possibility to obtain the acylated product instead of the alkylated-compound when the reaction was performed under aerobic conditions (Scheme 45) [55]. Indeed, the alkylated product **44o**–**t** can also exist under its keto–enol form, and will thus be oxidized by singlet oxygen, produced in situ from energy transfer between the photocatalyst and triplet oxygen. Electron-rich alkynes **53** were effective under the optimal conditions (**44u**–**y**), even though a slight modification of the procedure was necessary for diphenylethyne, as the reagent was oxidized in situ under the aerobic photoredox conditions. The introduction of functional groups on the pyridine *N*-oxide **35** diminished the yields (**44v-y**) compared to **44u**. It is worth mentioning that terminal alkynes were also subjected to this methodology with moderate success (**44z**).

**Scheme 46:**
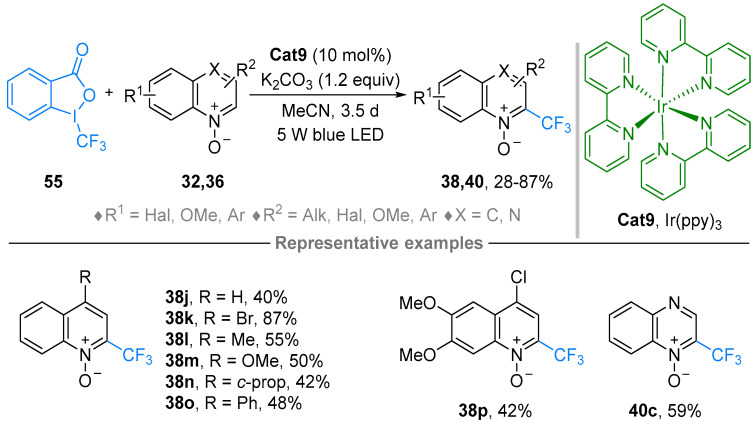
Photoredox-catalyzed reaction of azaarene *N*-oxides with Togni’s reagent.

By also taking advantage of photoredox catalysis, Gao and coworkers developed a new method to perform C2-trifluoromethylation of quinoline *N*-oxides **32** (Scheme 46) [56]. The very mild combination of Ir(ppy)_3_ and Togni’s reagent **55** allowed the retention of the *N*-oxide form at the end of the reaction. Electron-withdrawing groups (**38k**) on **32** provided better results than electron-donating ones (**38l**–**o**). Quinoxaline *N*-oxide **36** was also a potent substrate under these reaction conditions (**40c**).

**Scheme 47:**
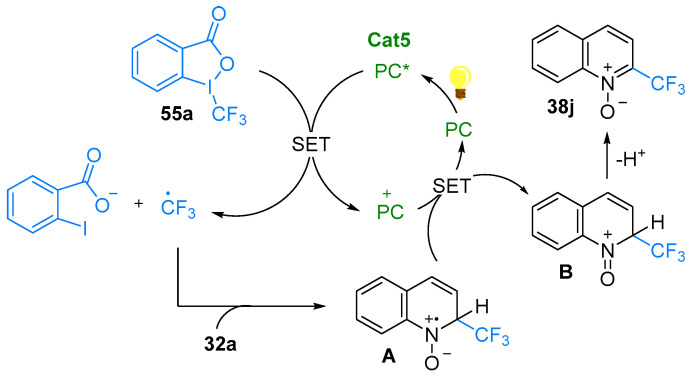
Proposed mechanism for the photoredox-catalyzed reaction of azaarene *N*-oxides with Togni’s reagent.

An oxidative quenching cycle was proposed by the authors, in which the excited photocatalyst reduced Togni’s reagent **55a** to generate the trifluoromethyl radical (Scheme 47). The quinoline *N*-oxide **32a** reacted with the radical to form the radical cation **A**. This intermediate was further oxidized by the photocatalyst, closing the catalytic cycle, and delivering the product **38j** after rearomatization by loss of a proton.

**Scheme 48:**
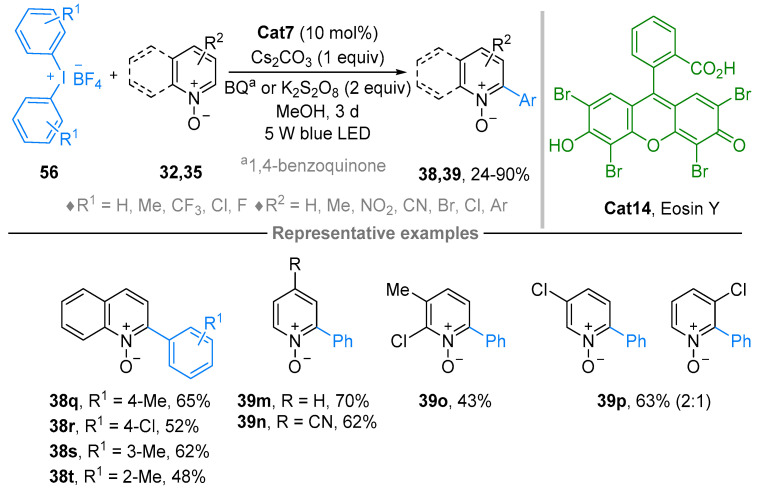
Photoredox-catalyzed reaction of azaarene *N*-oxides with diaryliodonium tetrafluoroborates.

In 2020, the groups of Song and Gao reported the C2 selective arylation of pyridine **35** and quinoline *N*-oxides **32** mediated by visible light catalysis and with diaryliodonium tetrafluoroborate **56** as the radical precursor (Scheme 48) [57]. Substitution on the arene ring of **55** did not affect the reaction (**38q**–**t**). In sharp contrast, substitution on the pyridine *N*-oxide **35** mattered, considering the poor regioselectivity when functional groups were introduced at the meta position (**39p**). The mechanism is similar to the one described above in Scheme 47, with the generation of the aryl radical by reduction of the iodonium reagent via single electron transfer from the photocatalyst.

**Scheme 49:**
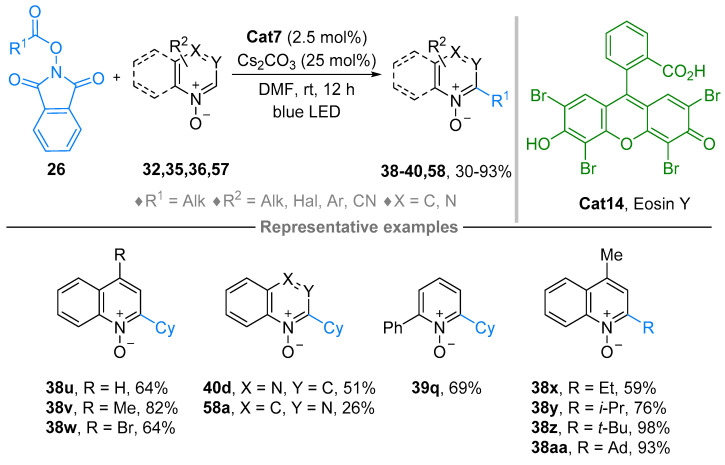
Photoredox-catalyzed reaction of azaarene *N*-oxides with NHPI redox-active esters.

Inspired by the abovementioned work and considering the appealing features of *N*-hydroxyphtalimide (NHPI), the group of Zhou described the synthesis of C2-alkylated heteroarenes *N*-oxides **32, 35, 36, 57** (Scheme 49) [58]. The reaction proceeded on quinoline (**38u-z,aa**), quinoxaline (**40d**), quinazoline (**58a**) and pyridine (**39q**) *N*-oxides. The selectivity for the C2 position was high (**38u,39q**) and it appeared that the more substituted the alkyl group, the higher the yield (**38x-z,aa**). The mechanism of this reaction will not be further described as it resembles the catalytic cycle in Scheme 47, with the initiation of the reaction via reduction and decarboxylation of NHPI **26** by the photocatalyst, as supported by Stern–Volmer experiments.

**Scheme 50:**
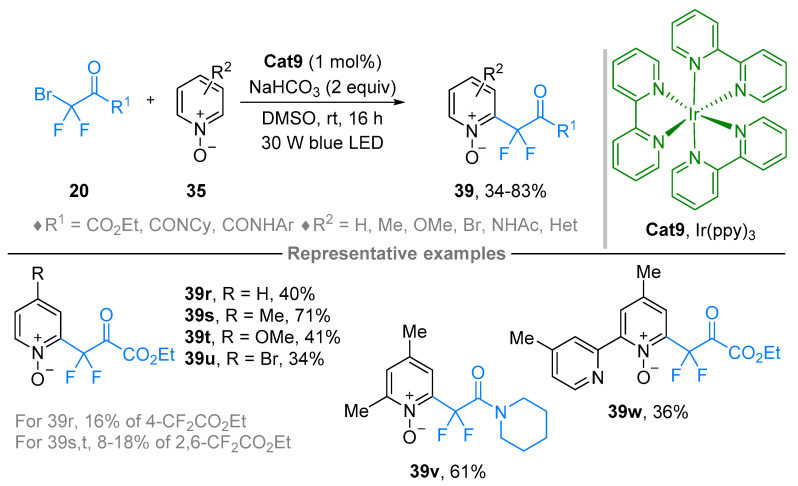
Photoredox-catalyzed reaction of azaarene *N*-oxides with bromo difluoro acetate derivatives.

In 2022, Wan and coworkers enlarged the field of C2 functionalization of heteroarene *N*-oxides **35** by developing a method to introduce selectively difluoro-acetate and -amide groups (Scheme 50) [59]. This was achieved under visible light-mediated conditions with *fac*-Ir(ppy)_3_ as the photocatalyst. It should be noted that the regioselectivity was moderate, as *para*-substituted side products were obtained (**39r**), as well as bis-*ortho*-functionalized compounds (**39s-u**). Interestingly, the reaction was selective for pyridine *N*-oxide over the non-oxidized pyridine ring (**39w**). In a similar fashion as the above-mentioned reactions, the mechanism is analogous to the one described in Scheme 47.

### 2.5. N-Aminopyridinium Ylides

**Figure 6:**
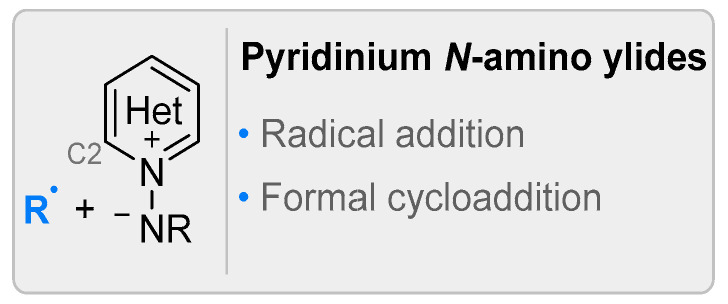
Radical-based transformations of pyridinium *N*-amino ylides.

In contrast to pyridinium *N*-oxides and *N*-aminopyridinium salts, *N*-aminopyridinium ylides have been much less described in the context of catalytic radical reactions [37,60] (Figure 6). Indeed, only two reports have been published since 2015 to the best of our knowledge. The regioselectivity follows the same as previously described for pyridine *N*-oxides, namely favored at sarbon-2.

**Scheme 51:**
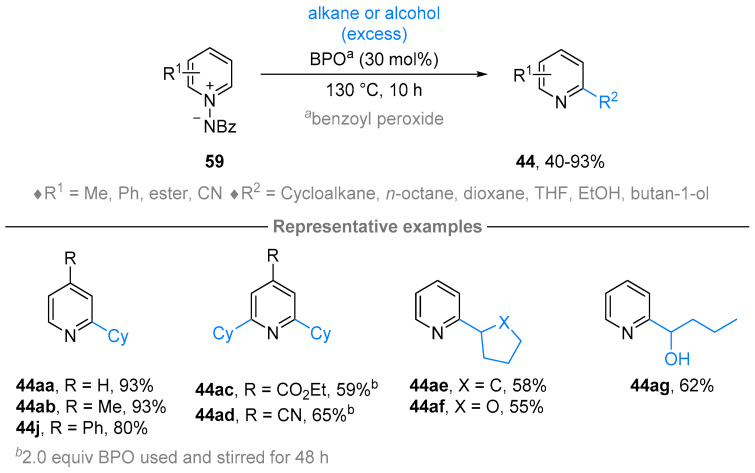
BPO-catalyzed alkylation of *N*-aminopyridinium ylides with alkanes or alcohols.

In 2019, the groups of Yu and Wang developed a new method to functionalize *N*-aminopyridinium ylides **59** at the *ortho* position, via a radical cross-dehydrogenative coupling (CDC) conducted under air, with benzoyl peroxide (BPO) as the radical initiator (Scheme 51) [61]. Several alkanes (**44aa**–**ae**) and alcohols (**44af**–**ag**) were successfully inserted with a high regioselectivity for the C2 position for most of the examples, an important asset when compared with Minisci or other CDC reaction conditions.

**Scheme 52:**
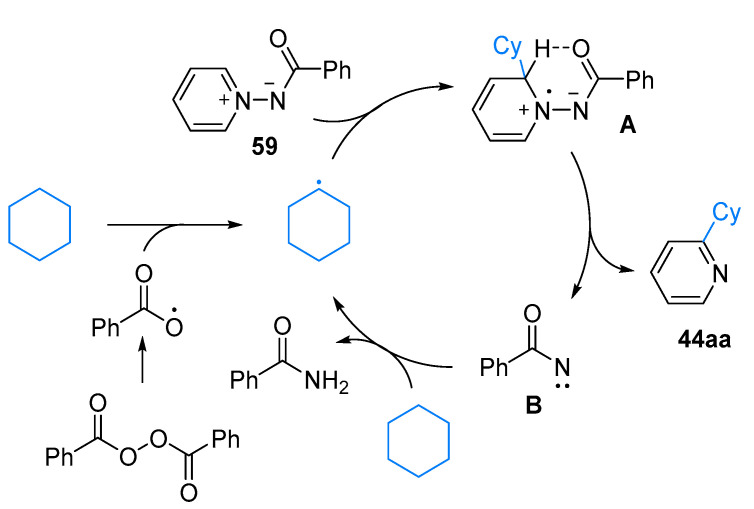
Proposed mechanism for the BPO-catalyzed alkylation of *N*-aminopyridinium ylides with alkanes or alcohols.

Based on the experimental results, the mechanism was described to start with the homolytic cleavage of the O–O bond of benzoyl peroxide, generating a carboxyl radical, which further abstracted a hydrogen atom from the alkane or the alcohol (Scheme 52). This new alkyl radical R^●^ was further trapped by *N*-aminopyridinium ylide **59** to form the radical cation **A**, which fell apart by cleavage of the N–N bond to deliver the desired product **44aa** and a nitrene intermediate **B**. The hydrogen bonding and subsequent formation of a six-membered ring in **A** clearly explained why the regioselectivity was high in this reaction. The latter abstracted hydrogen atoms from the alkane or the alcohol to release benzoic amide and regenerated the alkyl radical R^●^.

**Scheme 53:**
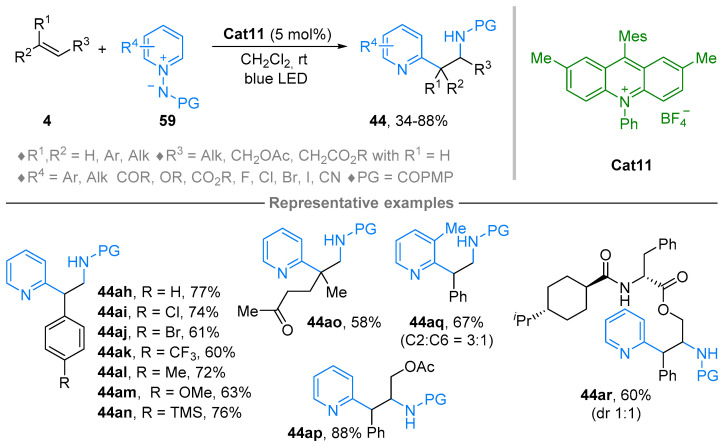
Photoredox-catalyzed reaction of *N*-aminopyridinium ylides with alkenes.

One year later, Hong and coworkers reported an elegant method to access C2-pyridyl *β*-amino motifs **44ah**–**ar** (Scheme 53) [62]. They were inspired by precedents on 1,3-dipolar cycloadditions of *N*-aminopyridinium ylides **59** with alkynes and envisaged a similar reaction with alkenes **4** followed by reductive cleavage of the N–N bond to deliver the desired product **44ah**–**ar**. Photochemistry was the key to overcome the endergonic barrier of these cycloaddition reactions with olefins by in situ generation of a *N*-radical pyridinium intermediate. With an acridinium derivative **Cat11** as the photocatalyst and upon blue LED irradiation, a wide range of mono- or disubstituted terminal alkenes (**44ah**–**ao**) as well as difunctionalized internal alkenes (**44ap**) were tolerated in this reaction, with the formation of only one diastereoisomer with a *syn* configuration. Substituted pyridines at the C2, C3 and C4 positions were successfully applied to this method (**44aq**) with excellent regioselectivity when the scaffolds were functionalized at the C2- and C4-postion. Furthermore, late-stage diversification of biorelevant molecules was conducted (**44ar**).

**Scheme 54:**
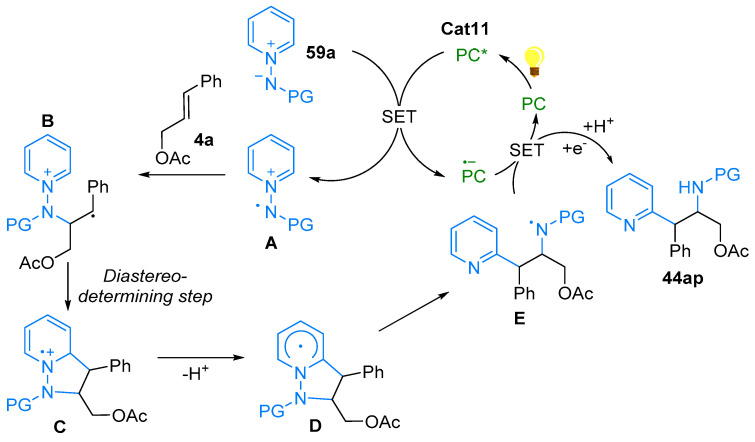
Proposed mechanism for the photoredox-catalyzed reaction of *N*-aminopyridinium ylides with alkenes.

DFT calculations were conducted to shed light on the reaction mechanism and the diastereo-determining step when diastereoisomers were formed (Scheme 54). After photoexcitation of the catalyst PC, the excited state PC* oxidized the *N*-aminopyridinium ylide **59a** (*E* = +1.58 V vs. SCE in MeCN) to form the radical cation intermediate **A**, which further reacted with alkene **4a** to deliver intermediate **B**. The formal radical-based cycloaddition reaction resulting in the bicyclic intermediate **C** was calculated as the diastereo-determining step, with the *syn* isomer being lower in energy. Next, deprotonation of intermediate **C** followed by homolytic cleavage of the N–N bond delivered intermediate **E**, the latter being fast and downhill in energy. A final step of reduction and protonation regenerated the catalyst and released the desired product **44ap**.

## 3. *N*-Alkylazaarenium Salts

**Figure 7:**
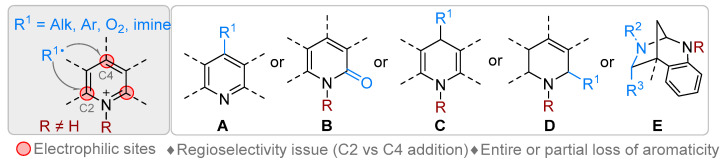
Radical-based transformations of *N*-Alkyl azaarenium salts.

*N*-Alkylazaarenium salts (i.e., pyridinium **60**, quinolinium **61** or isoquinolinium **62**) are important classes of substrates that have been involved in numerous reactions as electrophilic partners [63]. Thus, several nucleophiles, such as organometallic species (stoichiometric addition of Grignard reagents, metal-catalyzed additions of various reagent, etc.) [64] or acidic pro-nucleophiles under organocatalytic conditions (including asymmetric versions) to cite a few [65], were added to azaarenium salts providing access to 3D heterocycles from flat heteroaromatic structures. Although this field of research was intensively explored, some challenges remain: (1) generally speaking, azaarenium salts exhibit different electron-deficient sites (namely C2 or C4 positions for pyridines and quinolines), rendering the regioselective addition very challenging; (2) the addition of the nucleophilic species generally results in a transient dearomatized intermediate (namely dihydroazaarene derivatives), thus requiring high energy cost for the dearomatization step, especially if one considers pyridine ring, which requires a complete loss of aromaticity compared to quinolines or isoquinolines for which only a partial dearomatization occurs. Surprisingly, in this context, radical species have been less regarded as nucleophilic partners. Nevertheless, as described below such radical-based strategies met some successes in the synthesis of functionalized pyridines **A**, pyridinones derivatives **B**, 1,4-(and 1,2-)dihydroazaarenes **C**, tetrahydroazaarenes **D** and 1,3 diazepanes **E** thanks to either classical radical chemistry, modern photoredox catalysis or electrosynthesis (Figure 7).

**Scheme 55:**
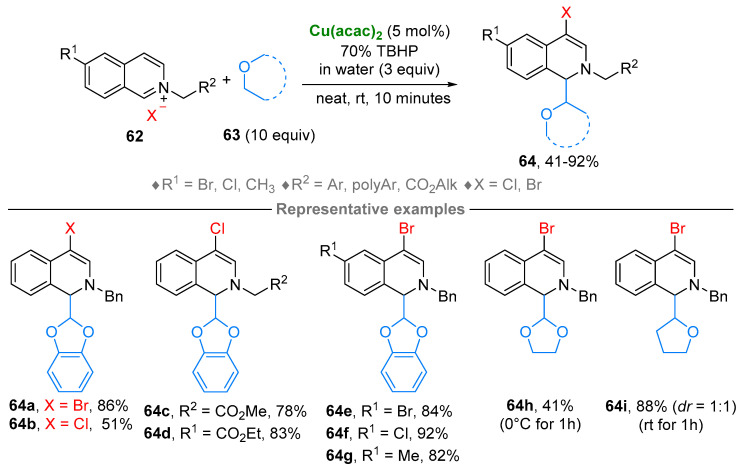
Cu(II)-catalyzed reaction of isoquinolinium salts with ether derivatives.

In 2018, Yan et al. reported on a copper-catalyzed 1,4-difunctionalisation of isoquinolinium salts **62** by an ether **63** and a halide to provide highly-functionalized dihydroisoquinolines **64** [66]. The reaction proceeded very smoothly under simple conditions (neat, rt for 10 min), affording generally high isolated yields for a large array of *N*-alkyl salts **62** (Scheme 55).

**Scheme 56:**
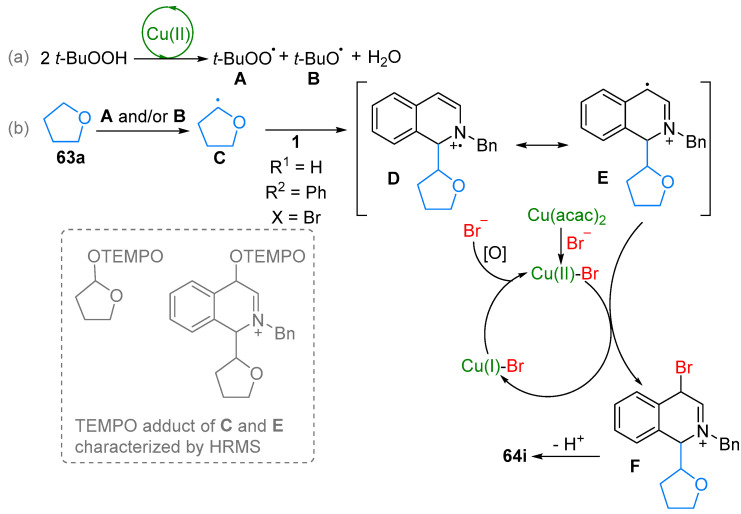
Proposed mechanism for the Cu(II)-catalyzed reaction of isoquinolinium salts with ether derivatives.

Bromine or chlorine atoms can be introduced at C4 position (R^2^ = Ph), albeit lower yields were obtained for the chlorinated product **64b** compared to brominated **64a**. It is worthy to note that some of the chlorinated products **64c** and **64d** can be obtained in good yields, provided that the phenyl substituent at R^2^ is replaced by an ester moiety. Substitution of the aromatic ring was also well-tolerated, providing **64e**–**g** without altering the isolated yields (84–92%). As far as the acetal part was concerned, aromatic acetal can be replaced by aliphatic ones but at the expense of a higher reaction time and lower temperature (1h at 0 °C instead of 10 min at rt) resulting in a decrease in yield for **64h**. Even THF can be introduced in high 88% isolated yield for **64i**. Nevertheless, longer reaction time was required and no diastereoselectivity was observed. Regarding the mechanism and taking molecule **64i** as example, the first step would be the homolytic decomposition of TBHP to generate radical intermediate **A** and **B** (Scheme 56a) followed by a hydrogen abstraction at the α position of the THF to provide a nucleophilic radical **C** (Scheme 56b) that added to the C2 electrophilic position of the isoquinolinium salt **62**, leading to the electrophilic *N*-centered radical cation **D**. This radical cation was in resonance with **E**, which underwent an oxidation step by Cu(II) to provide a likely di-cation intermediate that was trapped by a bromide to furnish the corresponding iminium **F**, which upon deprotonation led to the desired product **64**. One should note that radical intermediates **C** and **E** have been trapped by TEMPO and the adducts were characterized by HRMS.

**Scheme 57:**
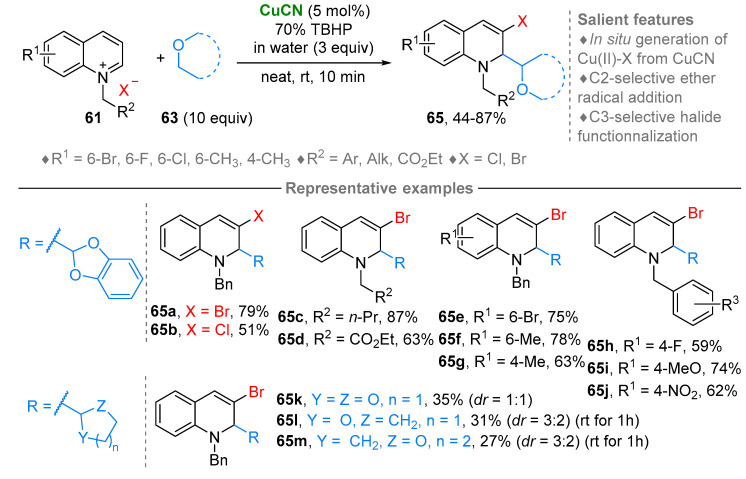
Cu(I)-catalyzed reaction of quinolinium salts with ether derivatives.

In 2019, the same group extended the methodology to the more challenging quinolinium salts **61** (Scheme 57) [67]. In this case, CuCN was found to outperform Cu(acac)_2_ in terms of isolated yield of the dihydroquinoline **65**. Under the optimized conditions, a large number of dihydroquinolines **65** were obtained in isolated yields ranging from 44% to 87% for the addition of aromatic acetals. In line with what has previously been observed (Scheme 55), the use of chloride salts instead of bromide resulted a drop of the yield of the corresponding dihydroquinoline (79% for **65a** vs. 51% for **65b**). Different substituents at the quinoline nitrogen (**65c**–**d**)—including a large panel of benzyl groups (**65h**–**j**)—and aromatic quinoline ring (**65e**–**g**) were well-tolerated without altering yields. The introduction of other ethers was more complicated and generally low isolated yields and dr (for aliphatic nonsymmetric ethers) were observed for compounds **65k**–**m**. In this case, two salient features were observed. On one hand, the addition of the ether radical occurred selectively at the C2 position. On the other hand, the halogenation took place selectively at C3 position while substitution of the C8 position was not observed (radical at C3 position obtained after the first addition of the ether radical is in resonance with the C8 position). This phenomenon was correlated by DFT calculations that exhibited a higher spin density at C3. The proposed catalytic cycle is almost the same as described in Scheme 56 except that the first step would be the in situ oxidation of CuCN to Cu(II)-Br.

Pyridones **66**, quinolones **67** and isoquinolinones **68** are an important class of compounds found in many natural or pharmaceutical products. Generally speaking, a two-step classical synthesis was developed consisting of the first synthesis of the *N*-Bn isoquinonilinium salt **62** followed by an oxidation step by an over-stoichiometric amount of K_3_Fe(CN)_5_, thus resulting in the formation of large amount of chemical waste [68]. Several research groups have turned their attention to the development of catalytic methods for the synthesis of such compounds. Nevertheless, to fulfill the scope of this review, approaches that involve the in situ generation of the isoquinolinium salts will not be discussed [69,70,71].

**Scheme 58:**
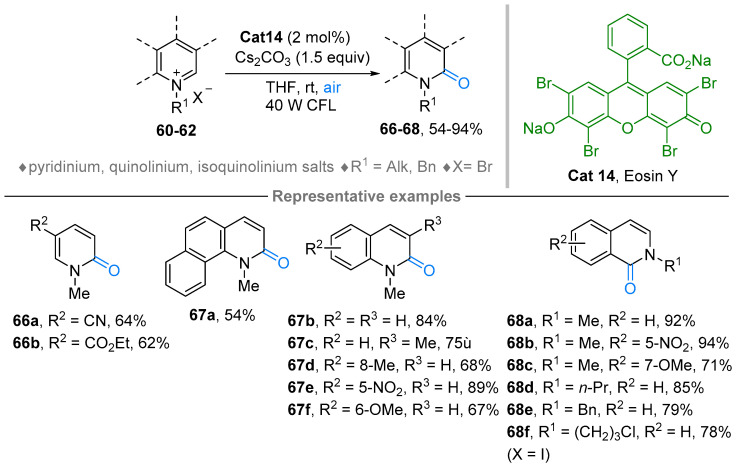
Photoredox-catalyzed aerobic oxidation of azaareniums salts in the presence of Eosin Y catalyst.

In that context, the group of Fu has reported on a visible light-mediated aerobic oxidation of azaarenium salts **60**–**62** under organic photocatalytic conditions (eosin Y (2 mol%), 40 W compact fluorescent light (CFL), Cs_2_CO_3_ (1.5 equiv.), air, THF, rt) (Scheme 58) [72].

**Scheme 59:**
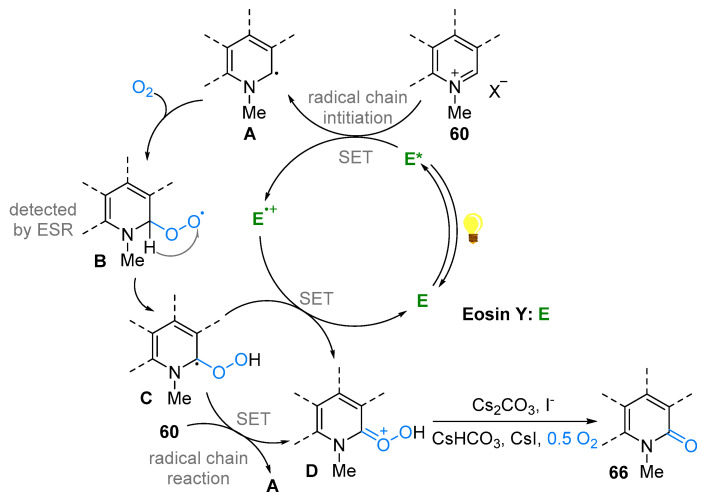
Proposed mechanism for the photoredox-catalyzed aerobic oxidation of azaareniums salts in the presence of Eosin Y catalyst.

Under standard conditions, a large panel of azaarenium salts (pyridinium **60**, quinolinium **61** and isoquinolinium **62**) were transformed into the corresponding dihydroazaarenones **66-68** in modest-to-excellent isolated yields. While the conditions exhibited a good functional group tolerance, the presence of electron-withdrawing groups on the aromatic ring (**67e** and **68b**) generally resulted in higher isolated yields compared to electron-donating groups (**67f** and **68e**). Several mechanistic experiments based on electron spin resonance (ESR), ^18^O labelling experiments, radical trapping, UV-Vis and NMR spectroscopies have been undertaken to shed light on the mechanism (Scheme 59). Several salient features were underlined: (1) the incorporated oxygen atom came from O_2_ rather than from H_2_O, (2) the reaction involved likely radical species, (3) single oxygen pathway was ruled out, (4) no EDA (electron donor–acceptor) complex was observed and (5) a radical chain process was implied. All these data allowed for the proposition of a plausible mechanism for the reaction. First, eosin Y (**Cat14**) was transformed into its excited state Cat14* thanks to irradiation under visible light. A SET process occurred to afford intermediate radical **A** and **Cat14^•+^** from **60**. Then, coupling of **A** with O_2_ provided the alkdioxyl radical **B** (detected by ESR), which upon hydrogen radical migration followed by another SET process delivered the oxygen-centered cation **D** with concomitant regeneration of the eosin Y catalyst. Meanwhile, a radical chain reaction via a SET process between **60** and **C** provided **A** and **D**. Finally, the oxidized product **66** was formed upon treatment.

**Scheme 60:**
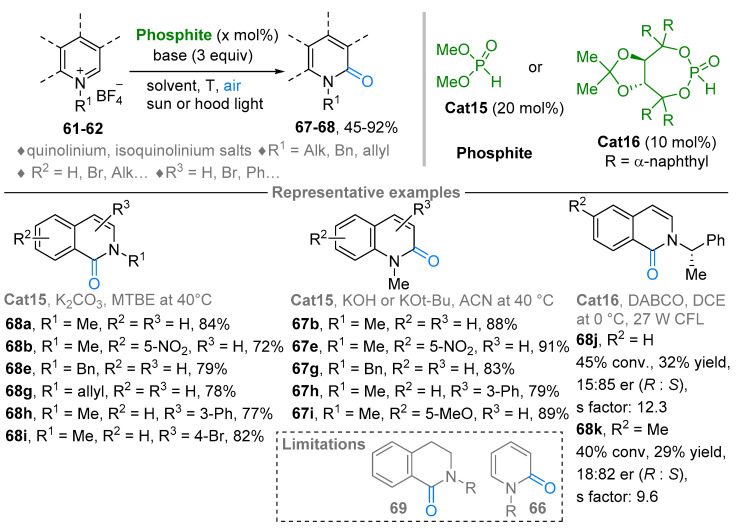
Photoredox-catalyzed aerobic oxidation of azaareniums salts in the presence of phosphite catalyst.

Organocatalysis, a well-established catalytic strategy recently awarded the Nobel price of chemistry, has also been involved in the aerobic oxidation of azaarenium salts under photochemical conditions. In 2018, Maity and coworkers reported on the use of dimethyl phosphite organocatalyst (**Cat15**, 20 mol%) for the aerobic oxidation of a wide panel of quinolinium **61** and isoquinolinium salts **62** in the presence of three equivalents of a base (either K_2_CO_3_, KOH or KO*t*-Bu) in acetonitrile or MTBE depending upon the substrates at 40 °C (Scheme 60) [73]. Under air and light irradiation (sunlight or hood light, no reaction in the dark), an efficient reaction took place thus leading to the corresponding (iso)quinolones **67** and **68** with generally high isolated yields (26 examples, 47–92%) for a wide variety of substituents (alkyl, electron-donating or -withdrawing groups both on the nitrogen-containing ring or on the aromatic carbocyclic part). One should note that products **66** and **69** were not observed, as the corresponding pyridinium salts or cyclic iminiums precursors were reluctant under optimized conditions. The authors have also successfully developed an oxidative kinetic resolution reaction using chiral phosphite **Cat16** derived from TADDOL using DABCO as a base in DCE at 0 °C. Compound **68j** (R^2^ = H) was obtained in 70% ee (45% conversion, s factor 12.3) and product **68k** (R^2^ = Me) in 64% ee (40% conversion, s factor 9.6).

**Scheme 61:**
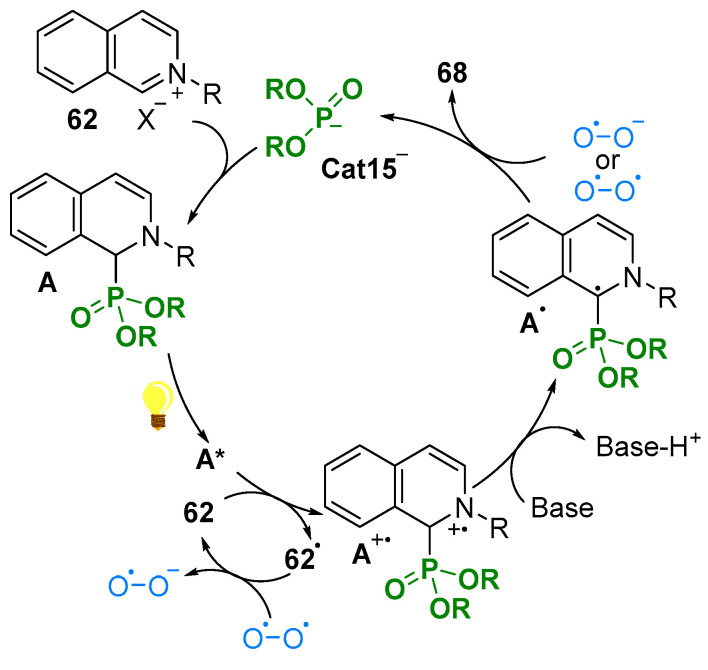
Proposed mechanism for the photoredox-catalyzed aerobic oxidation of azaareniums salts in the presence of phosphite catalyst.

Taking isoquinolinium salt **62** as a model and based upon UV-VIS measurements (no adsorption of **62** but adsorption of the adduct **A** and Stern–Volmer quenching experiments indicating a concentration and time-dependent decrease in fluorescence in the presence of **62**), the following mechanism was proposed (Scheme 61). At first, deprotonated phosphite catalyst **Cat15^−^** was added to **62** to provide the adduct **A** that, upon visible light excitation, underwent a SET process with the starting **62** to afford the corresponding radical **62^•^** and the radical cation **A^+•^**. The C–H acidity of the latter species was greatly increased affording the deprotonation via a base to provide the corresponding radical **A^•^**. Simultaneously, **62^•^** transferred one electron to O_2_ to regenerate **62** and to produce superoxide (O_2_**^−•^**). Finally, **A^•^** reacted with either superoxide (O_2_**^−•^**) or oxygen to afford the desired product **68** with concomitant regeneration of the deprotonated phosphite catalyst **Cat15^−^**.

**Scheme 62:**
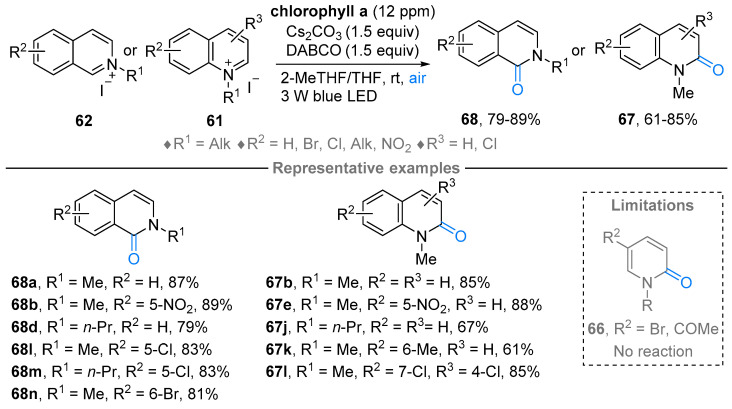
Photoredox-catalyzed aerobic oxidation of azaareniums salts in the presence of chlorophyll a catalyst.

In 2021, the group of Yadav has reported on the use of chlorophyll a (**Chla**) as a photocatalyst for the synthesis of quinolinones **67** and isoquinolinones **68** from the corresponding quinolinium **61** and isoquinolinium salts **62**, respectively, by a selective photo-induced electron transfer (PET) and energy transfer (EnT) process (Scheme 62) [74]. Under the optimized conditions (**Chla** (12 ppm), Cs_2_CO_3_ (1.5 equiv), DABCO (1.5 equiv), 3 W blue LED, 2MeTHF/THF, rt, air), the reaction proved to be efficient (61–89%) even if a narrow scope was reported (12 examples). Both quinolones **67** and isoquinolones **68** tolerate the presence of halogen, methyl or nitro group without altering the isolated yield. The alkyl chain on the nitrogen can also be lengthened but at the expense of the isolated yields which dropped from 87% to 79% for the isoquinolone series (**68a**, R = Me vs. **68d**, R = *n*-Pr) and from 85% to 67% in the quinolinone series (**67b**, R = Me vs. **67g**, R = *n*-Pr). Nevertheless, this phenomenon has not been observed when the aromatic ring was substituted by a Cl at position 5 (83% for **68l** and **68m**). As for the previous work of Maity et al. [73], under these conditions, pyridinium salts remained unreactive and the corresponding pyridinone compounds **66** were not observed. The mechanism proposed by the authors is the same as the one described by the group of Fu except that chlorophyll a was used as a photocatalyst instead of eosin Y (see Scheme 59).

**Scheme 63:**
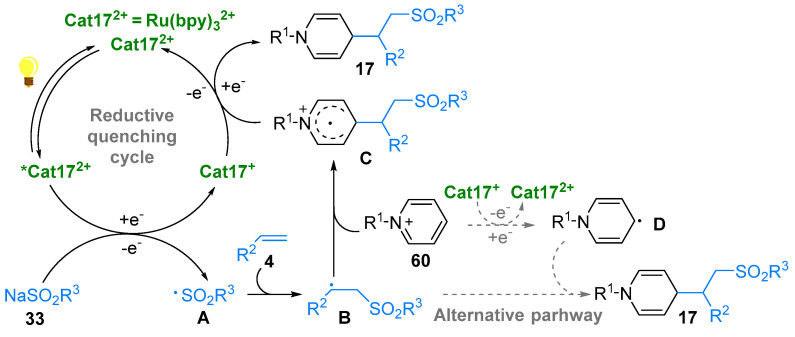
Proposed mechanism for the photoredox-catalyzed reaction of azaareniums salts with alkenes.

In 2020, Li and coworkers described a visible light-induced dearomatization reaction of pyridinium **60**, quinolinium **61** and isoquiniolinium **62** salts by a three-component approach involving a sodium sulfinate **33** and an alkene **4** [75]. The first step of this transformation was the formation of a sulfonyl radical **A** upon oxidation of the sodium sulfinate **33** by the excited Ru-photocatalyst (*Ru(bpy)_3_^2+^ = *Cat17^2+^) (Scheme 63). This sulfinate radical **A** then added to the alkene partner **4** to generate a new radical **B** that was nucleophilic enough to add to the pyridinium salt **60** at the position C4 exclusively to provide a radical cation **C** which, upon reduction by the reduced Ru-photocatalyst (Ru(bpy)_3_^+^ = **Cat17^+^**) thanks to a SET process, afforded the desired 1,4-dihydropyridine **17**. An alternative mechanism, involving the generation of a carbon-centered radical **D** generated by the reduction in the pyridinium salt (by the reduced Ru-photocatalyst, Ru(bpy)_3_^+^ = **Cat17^+^**) followed by a radical coupling with **B** to provide **17** cannot totally be ruled out, as traces of pyridine derivatives have been observed using GC-MS analysis.

**Scheme 64:**
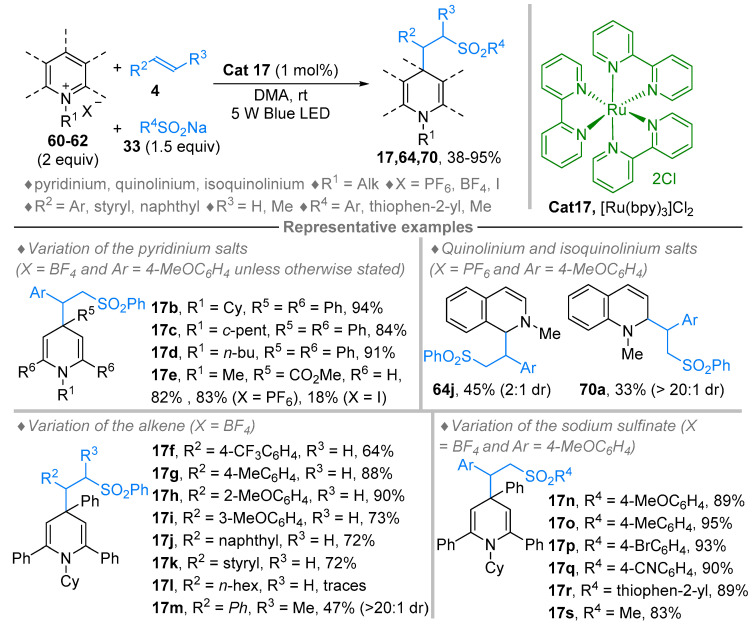
Photoredox-catalyzed reaction of azaareniums salts with alkenes.

Thus, by applying this strategy (**Cat17** (1 mol%), 5 W blue LED, DMA, rt), more than 30 substituted dihydroazaarenes **17, 64, 70** were obtained in isolated yields ranging from 38% to 95% starting from the corresponding azaarenium salts **60**–**62** (Scheme 64). Regarding the substitution of the 1,4-dihydropyridine **17**, several alkyl groups (**17b** (Cy, 94%), **17c** (*c*-pent, 84%), **17b** (*n*-Bu, 91%)) can be introduced on the nitrogen atom without significant influence on the yields. A 2,4,6-triphenyl substitution pattern (**17b**–**d**) was well-tolerated as well as C4-substitution by a CO_2_Me group (**17e**). The nature of the anion was demonstrated to be crucial as I^−^ (18% yield) provided significant drop in the yield compared to BF_4_ or PF_6_ anions (82% and 83%) for **17e**. The substitution of alkene partner **4** has also been studied. Whereas 4-MeO phenyl **17b** provided the best result in terms of isolated yield (94%), other substituents on the aromatic ring of the alkene partner (**17f** (4-CF_3_, 64%), **17g** (4-Me, 88%), **17h** (2-OMe, 90%), **17i** (3-OMe, 73%)) have also been successfully introduced. Naphthyl or styryl gave the corresponding dihydropyridines **17j** and **17k** in 72% and 75% yields, respectively. The alkene with a substituent at the β-position provided the desired product **17m** albeit in modest a 47% isolated yield but high diastereoselectivity (>20:1 dr). It is worthy of note that alkene with an aliphatic substituent (**17l**, *n*-hexyl) was not reactive under the standard conditions. The sodium sulfinate **33** can also be diversely substituted. Aryl sodium sulfinates with *para* substituent (OMe, Me, Br, CN), thiophen-2-yl sodium sulfinate or methyl sodium sulfinate were also eligible for this reaction providing the corresponding desired products **17n**-**s** in isolated yield above 70%. Moving from pyridinium salts **60** to quinolinium **61** or isoquinolinium salts **62** resulted in a drop of the isolated yields in the corresponding dihydroisoquinoline **64j** (45%, 2:1) and dihydroquinoline **70a** (33%, >20:1).

**Scheme 65:**
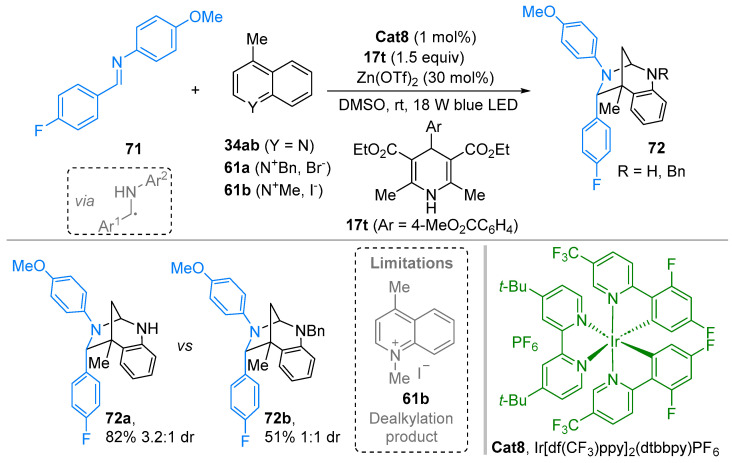
Photoredox-catalyzed reaction of quinolines and quinolinium salts with imines.

In 2020, the group of Dixon reported the dearomatization of quinoline **34** by reaction with imines **71** under photoredox conditions (blue LED and Ir photocatalyst **Cat8**) to provide bridged 1,3-diazepanes **72** (Scheme 65) [76]. During the study of the reaction, only one example was reported using the more activated *N*-benzyl quinolinium salt **61a** derived from lepidine. The *N*-benzyl-1,3-diazepane product **72b** was obtained albeit with significant loss in both yield and diastereoselectivity compared to the 1,3-diazepane **72a** (51%, 1.0:1.0 dr vs. 82%, 3.2:1.0 dr, respectively). The *N*-methylledipidium salt **61b** was reluctant to the photochemical conditions, thus resulting in the formation of the dealkylation product. Extensive mechanistic study was performed to explain the formation of *N*-H 1,3-diazepanes **72**; in particular, the role of the Lewis acid (Zn(OTf)_2_) was demonstrated to allow the imine partner **71** to be more easily reduced than the quinoline derivative **34**. The coordination of the Lewis acid to the imine partner **71** rather than to the quinoline **34** was also supported by the fact that Lewis acid was still beneficial for the reaction with quinolinium salt **61**. Nevertheless, no mechanism was reported for the specific case of quinolinium salt **61**.

**Scheme 66:**
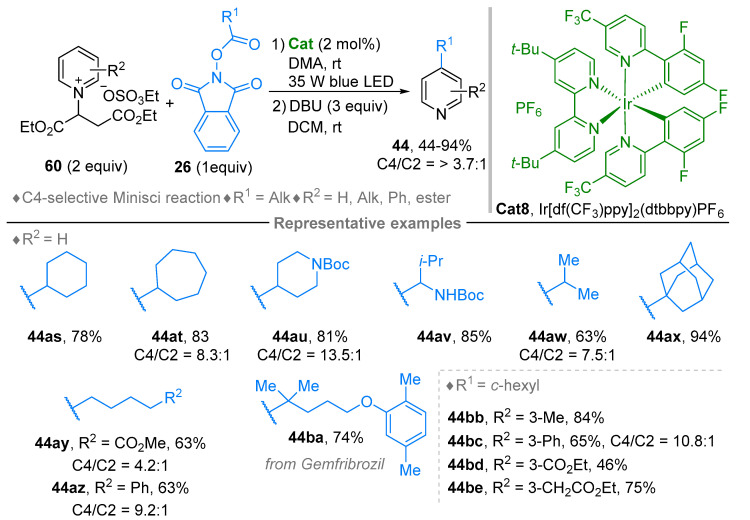
Photoredox-catalyzed reaction of pyridinium salts with NHPI redox-active ester.

In 2021, Baran et al. have described a Minisci reaction using malonate-derived substituents on the pyridine nitrogen as a blocking group to afford C4 selective alkylation of the pyridine in the presence of two equivalents of ammonium persulfates as a chemical oxidant and two equivalents of carboxylic acid as radical precursor [77]. Two years later, Zhang, Yang and coworkers published an improved procedure using a photoredox-catalyzed approach that avoided the use of more than a stoichiometric amount of oxidant under neutral conditions (use of NHPI-derived redox-esters **26** as radical precursor instead of a carboxylic acid) [78]. Under standard conditions (iridium catalysts (1 mol%), 35 W blue LED, DMA, rt), several alkylated pyridines **44** were obtained after removal of the blocking group (DBU, DCM, rt) in moderate-to-excellent isolated yields (44–94%) (Scheme 66). Interestingly, the presence of the malonate-derived blocking group on the nitrogen revealed to be crucial for the control of the regioselectivity of the radical addition, thus providing a good-to-excellent C4/C2 ratio (>3.7:1). Primary radicals were well-tolerated (**44ay**–**44az**) even if the C4/C2 ratios were lower than those observed with secondary (**44as**–**aw**) or tertiary radicals (**44ax** and **44ba**), with the tertiary radical providing the C4 adduct exclusively. It is worth noting that drug molecules such as Gemfibrozil (via its corresponding NHPI) can be straightforwardly prepared through this methodology (**44ba**, 74%). The substitution pattern of the pyridine ring was also tackled during the introduction of a cyclohexyl pendant at C4 position (R^1^ = Cy). Methyl (**44bb**) and CH_2_CO_2_Et (**44be**) at C3 position provided the desired product in high yields (84% and 75%) and complete C4 regioselectivity, whereas CO_2_Et (**44bd**) and to a lesser extent Ph (**44bc**, in addition to a drop of the C4/C2 ratio to 10.8:1) provided significantly lower yields of 46% and 65%.

**Scheme 67:**
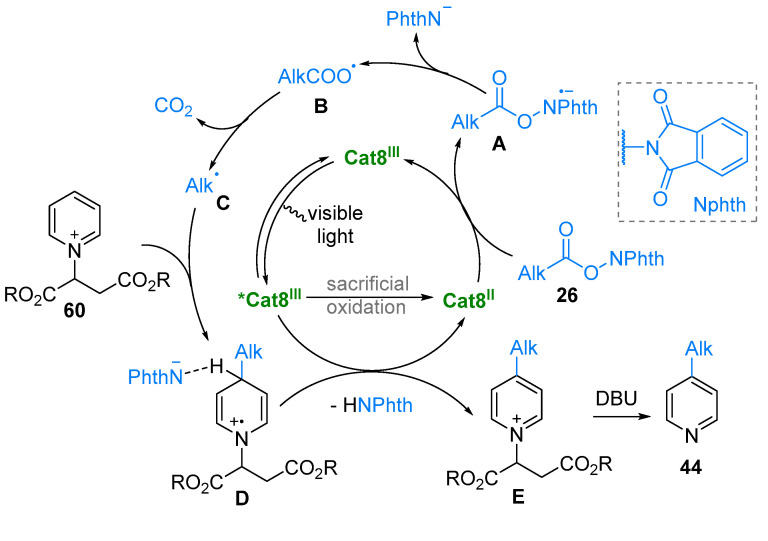
Proposed mechanism for the photoredox-catalyzed reaction of pyridinium salts with NHPI redox-active ester.

The first step of the mechanism was considered to be the formation of *Cat8^III^ by light irradiation of **Cat8^III^** (Scheme 67). Then, **Cat8^II^** might be generated from *Cat8^III^ using the solvent as a sacrificial oxidant or an anion generated from the decomposition of NHPI esters. The NHPI derivative **26** could then be reduced by **Cat8^II^** to the corresponding radical anion **A** via a SET pathway followed by elimination of a phthalimidate ion to provide the radical **B**, which upon decarboxylation finally afforded the alkyl radical **C**. Then, the latter radical was added regioselectively at the C4 position of the pyridinium salt **60**, giving rise to the corresponding *N*-centered radical cation **D**. *Cat8^III^ would then oxidize the radical cation **D** to the pyridinium **E** by concomitant deprotonation via the phthalimidate ion and subsequent regeneration of **Cat8^II^**. The desired alkylated pyridines **44** were obtained by treatment with DBU.

**Scheme 68:**
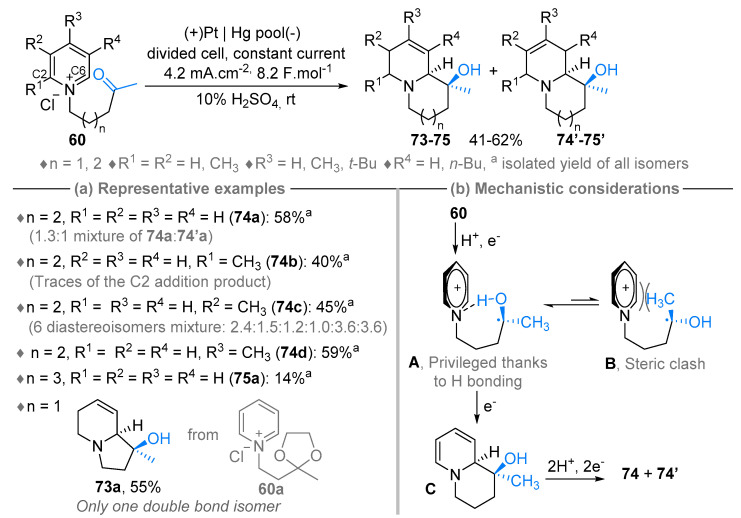
Intramolecular electrochemical reaction of oxoalkyl pyridinium salts: (a) Representative examples (b) Mechanistic considerations.

Although electrosynthesis has a long-standing history, this technique has faced a revival at the beginning of the 2010′s in the quest for greener processes (replacement of stoichiometric amount of generally toxic oxidant/reductant by simple electrons). It is not surprising that an electrochemical dearomatization of pyridinium was reported by Schäfer et al. as early as 1995, which is worthy of note as a pioneering contribution. Indeed, the authors developed a cathodic cyclization of several oxoalkyl pyridinium salts **60** to provide the corresponding heterocycles **73** (*n* = 1), **74** (*n* = 2) and **75** (*n* = 3) (Scheme 68) [79].

Under galvanostatic conditions (constant current), several quinolizidines and indolizidines were obtained as a mixture of inseparable double bond isomers **74**–**74′** and **75**–**75′**, respectively, in moderate isolated yields ranging from 41 to 62% (Scheme 68a). For unsubstituted pyridinium salts, product **74a** was obtained as a 1.3:1 ratio of **74a** vs. **74′a**. The substitution of the *ortho* position of pyridinium salt by a methyl group (R^1^ = CH_3_) resulted in the selective addition at C6 position (only traces of C2 addition was observed) to provide the corresponding **74b** in 40% yield. The substitution of the *meta* position (R^2^ = CH_3_) of pyridinium salt provided the corresponding quinolizidines **77c** in moderate 45% yield as a complex mixture of six diastereoisomers. The substitution of the *para* position (**74d**) delivered similar results to those obtained for unsubstituted product **74a**. The seven-membered ring **75** (*n* = 3) was also obtained but at a very low yield of 14%. Indolizidine derivative **73a** was also accessible, provided that an acetal precursor **60a** was used instead of the ketone. In this case, **73a** was isolated in a 55% yield as a single double-bond isomer. Regarding the mechanism (Scheme 68b), a radical would be obtained after a first protonation/reduction sequence. Among the two conformations **A** and **B** of the radical, conformation **A** would be privileged thanks to hydrogen bond between the hydroxy and the nitrogen groups (AM1 calculation revealed that nitrogen in the radical cation displays a partial negative charge), whereas steric clash would disfavor conformation **B**. Then, a further SET reduction process provided dihydropyridine derivatives **C**, which upon further protonation/reduction sequence delivered **74** and **74′**. Worthy to note, the configuration of **74a** was proven by X-ray analysis.

## 4. Conclusions

Numerous examples from the recent literature have testified how modern catalytic and technology-driven strategies facilitate the implementation of radical-based chemistry in a more convenient fashion while expanding the scope of numerous synthetic methodologies. This impetus in the use of highly reactive radical species allowed the investigation of novel reactivity profiles and functional group transformations based on better controlled single-electron transfer events. In this review, we have highlighted the advantage, complementarity and originality of employing *N,N*-disubstituted iminium motif instead of more regular imine or protonated iminium salt derivatives in radical transformations. On the one hand, dipole-type iminium derivatives proved to be highly versatile platforms, with enhanced reactivity profiles in radical addition reactions, thereby allowing annulation, cycloaddition or transposition reactions to take place, especially with azaarenium salts for the latter. Additionally, the *N*-Alkylazaarenium structures afford new opportunities for the dearomatization sequences opening routes to valuable Csp^3^-rich azaheterocycles. Nevertheless, one can consider that the regioselectivity of the radical species additions, while being improved, are still a matter of investigation for azaarenium platforms. Electrosynthesis and electrocatalysis, regardless of the approach employed, remained underexploited in this type of chemistry in comparison to photoredox and metal-based catalysis, and will undoubtedly face important developments in the near future. Most importantly, the development of asymmetric syntheses in this field of research still remains in its infancy, leaving important room for novel investigations. We do hope this overview of recent examples in that field of investigations will serve as a tool to trigger novel developments and applications on radical-based transformations of *N*-tertiary iminium derivatives.
